# Conformational Changes in a Hyperthermostable Glycoside Hydrolase: Enzymatic Activity Is a Consequence of the Loop Dynamics and Protonation Balance

**DOI:** 10.1371/journal.pone.0118225

**Published:** 2015-02-27

**Authors:** Leandro C. de Oliveira, Viviam M. da Silva, Francieli Colussi, Aline D. Cabral, Mario de Oliveira Neto, Fabio M. Squina, Wanius Garcia

**Affiliations:** 1 Departamento de Física, Instituto de Biociências, Letras e Ciências Exatas, UNESP—Univ Estadual Paulista, São José do Rio Preto, SP, Brazil; 2 Centro de Ciências Naturais e Humanas, Universidade Federal do ABC (UFABC), Santo André, SP, Brazil; 3 Departamento de Física e Biofísica, Instituto de Biociências, UNESP—Univ Estadual Paulista, Botucatu, SP, Brazil; 4 Laboratório Nacional de Ciência e Tecnologia do Bioetanol, Centro Nacional de Pesquisa em Energia e Materiais, Campinas, SP, Brazil; Oak Ridge National Laboratory, UNITED STATES

## Abstract

Endo-β-1, 4-mannanase from *Thermotoga petrophila* (TpMan) is a modular hyperthermostable enzyme involved in the degradation of mannan-containing polysaccharides. The degradation of these polysaccharides represents a key step for several industrial applications. Here, as part of a continuing investigation of TpMan, the region corresponding to the GH5 domain (TpManGH5) was characterized as a function of pH and temperature. The results indicated that the enzymatic activity of the TpManGH5 is pH-dependent, with its optimum activity occurring at pH 6. At pH 8, the studies demonstrated that TpManGH5 is a molecule with a nearly spherical tightly packed core displaying negligible flexibility in solution, and with size and shape very similar to crystal structure. However, TpManGH5 experiences an increase in radius of gyration in acidic conditions suggesting expansion of the molecule. Furthermore, at acidic pH values, TpManGH5 showed a less globular shape, probably due to a loop region slightly more expanded and flexible in solution (residues Y88 to A105). In addition, molecular dynamics simulations indicated that conformational changes caused by pH variation did not change the core of the TpManGH5, which means that only the above mentioned loop region presents high degree of fluctuations. The results also suggested that conformational changes of the loop region may facilitate polysaccharide and enzyme interaction. Finally, at pH 6 the results indicated that TpManGH5 is slightly more flexible at 65°C when compared to the same enzyme at 20°C. The biophysical characterization presented here is well correlated with the enzymatic activity and provide new insight into the structural basis for the temperature and pH-dependent activity of the TpManGH5. Also, the data suggest a loop region that provides a starting point for a rational design of biotechnological desired features.

## Introduction

The lignocellulosic biomass consists of cellulose in close association with hemicellulose and lignin [1–3]. The hemicellulose includes a variety of polysaccharides with linear or branched polymers derived from sugars such as D-xylose, D-galactose, D-mannose, D-glucose, and L-arabinose [4]. Classes of hemicellulose are named according to the main sugar unit, and most of the main-sugars on hemicellulose structure are linked together by β-1, 4-glycosidic bonds [4–6]. The hemicelluloses are estimated to account for one third of all components available in plants and are the second heteropolymers present in nature [4,7]. The biodegradation of hemicellulose polymer involves the concerted action of a variety of hydrolytic enzymes. The major enzymes involved in the biodegradation of mannan polysaccharides are β-mannanase (EC 3.2.178), β-mannosidase (EC 3.2.1.25), and β-glucosidase (EC 3.2.1.21) [7,8].

Archaea and bacteria isolated from harsh environments such as thermal hot springs and deep-sea hydrothermal vents are an excellent source of extremophilic enzymes. *Thermotoga petrophila* strain RKU-1 is a hyperthermophilic bacterium isolated from the production fluid of the Kubiki oil reservoir in Niigata (Japan), which grows optimally at pH 7 and 80°C [9]. This bacterium produces a repertoire of hyperthermostable enzymes of great industrial interest, including cellulases, arabinofuranosidases, arabinases and mananases, and it has proved to be a suitable source of enzymes for biotechnological applications and protein engineering of glycoside hydrolases [10–13].

The endo-β-1, 4-mannanases (EC 3.2.1.78) are enzymes that catalyze the hydrolysis of β-1, 4-mannoside linkages in a variety of mannose-containing polysaccharides, including glucomannans and galactomannans [7,10]. These enzymes have been widely studied owing to their roles in seed germination and fruit ripening [7]. However, in the last decade they have become biotechnologically important due to their ability to reduce complex polysaccharides into simple molecules such manno-oligosaccharides and mannoses. Their industrial applications include biobleaching of softwood pulps [14–16], extraction of oil from coconut meat [7,16], reduction of the viscosity of coffee extract [16], improvement of the nutritional value of livestock feeds [16], use as an additive in the detergent industry [8,16], and production of second-generation biofuels [6,16]. For agro-industrial purposes, the use of enzymes with a high stability under a broad range of pH and temperature is highly desirable [16].

The hyperthermostable bacterial endo-β-1, 4-mannanase (EC 3.2.1.78) from *T*. *petrophila* (TpMan) is an enzyme composed of a GH5 catalytic domain (TpManGH5) and a carbohydrate-binding domain (TpManCBM27) connected through a linker [10,13]. The structure of TpManGH5 is a canonical (α/β)_8_-barrel scaffold surrounded by loops and short helices that form the catalytic interface [10]. In a recent study, we presented the low-resolution model of the intact TpMan studied by small angle X-ray scattering [13]. Here, as part of a continuing investigation of TpMan, TpManGH5 was analyzed using biophysical techniques as well as molecular dynamics simulations. The biophysical characterization presented here is well correlated with the enzymatic activity and provide new insight into the structural basis for the temperature and pH-dependent activity of the TpManGH5.

## Results

### Influence of pH and Temperature on the Enzymatic Activity of the TpManGH5

**Fig 1:**
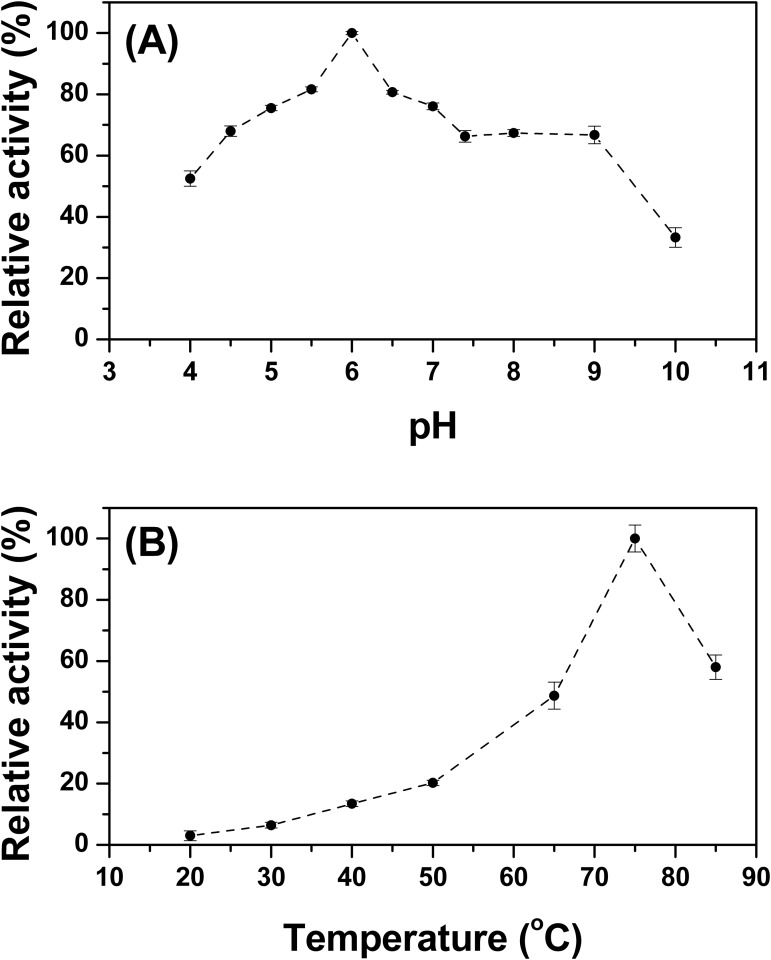
Enzymatic activity. (**A**) Effect of pH on the enzymatic activity of TpManGH5 at 75°C. (**B**) Effect of temperature on the enzymatic activity of TpManGH5 at pH 6. The experimental conditions are described in Materials and Methods.

The activity of an enzyme is dependent on pH and temperature, conditions that invoke changes in enzyme folding, conformation, and protonation [17]. As a first step of our studies, the mannan endo-1, 4-β-mannosidase activity of TpManGH5 was evaluated as a function of pH using carob galactomannan as substrate (Fig. 1A). Our results showed that, under the conditions of our study (at 75°C), the activity of TpManGH5 increases as pH increases within the pH range of 4–6, decreases within the pH range of 6–8, remains approximately constant within the pH range of 8–9 and then decreases within the pH range of 9–10 (Fig. 1A). TpManGH5 reaches its optimum activity at pH 6 and 75°C. At pH 4 and 10, it was found that the remaining activity of the TpManGH5 was 53% and 33%, respectively. At alkaline pH values between 7 and 9 TpManGH5 showed remaining activity constant around 70%. At pH levels below 3 (highly acidic pH) and above 10 (highly alkaline pH) the remaining activities were virtually zero (data not shown). Next, the mannan endo-1, 4-β-mannosidase activity of TpManGH5 was studied as a function of temperature at pH 6 (Fig. 1B). In this experiment, TpManGH5 was initially incubated during 30 minutes in each temperature and subsequently the remaining activity was determined against carob galactomannan. Within the temperature range of 20 and 75°C (pH 6), the enzymatic activity of TpManGH5 increased with the increase in temperature, reaching its maximum at 75°C (Fig. 1B). However, at 85°C the enzymatic activity of TpManGH5 decreased significantly.

### Influence of pH on the Structural Stability and Global Compactness of the TpManGH5

**Fig 2:**
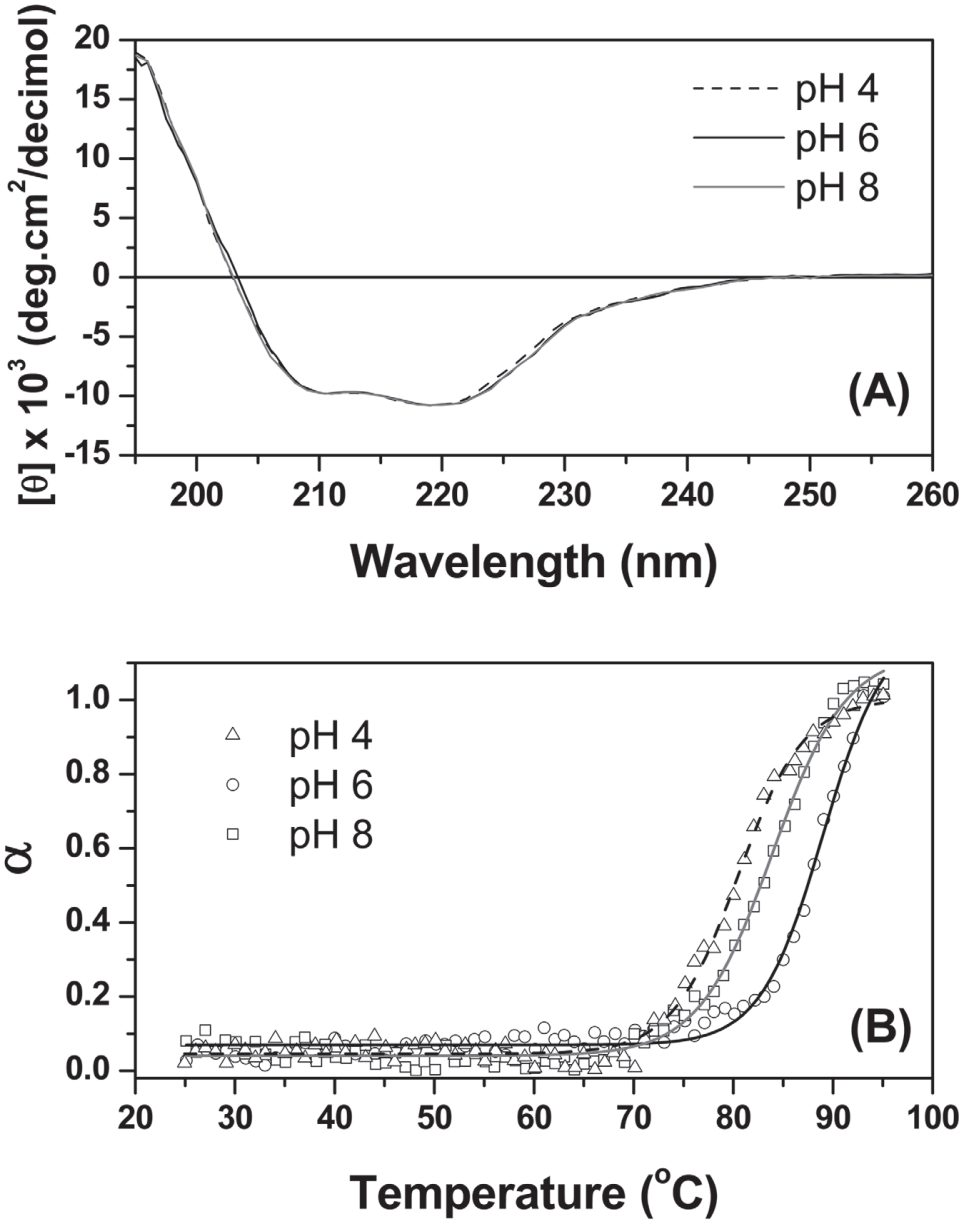
Circular dichroism (CD) spectroscopy. (**A**) Effect of pH on the secondary structure of TpManGH5 at 20°C monitored by far-UV CD spectroscopy. The pH values were 4 (black dash line), 6 (black solid line) and 8 (gray solid line). (**B**) Thermal denaturation of TpManGH5 was monitored by measuring the ellipticity at 220 nm as a function of temperature. The pH values were 4 (triangles), 6 (circles) and 8 (squares). The experimental conditions are given in Materials and Methods section.

Influence of pH on the structural stability and global compactness of the recombinant TpManGH5 was studied using far-UV circular dichroism (CD) spectroscopy and small-angle X-ray scattering (SAXS). Far-UV CD spectroscopy was used to probe the influence of pH on the secondary structure of the TpManGH5 (Fig. 2). The CD spectra for TpManGH5 collected at the three pH values and 20°C is shown in Fig. 2A. The CD spectrum of the TpManGH5 at pH 6 (black solid line) is characterized by a positive band at 195 ± 1 nm and two negative bands at 210 ± 1 nm and 220 ± 1 nm, together with a negative to positive crossover at 203 ± 1 nm [13]. These bands are typically found in structures with a large content of α-helical secondary structure. The far-UV CD spectra of the TpManGH5 at pH 4 and 6 were very similar to pH 6 (Fig. 2A). The results shown in Fig. 2A, therefore, indicate that the secondary structure of the TpManGH5, at 20°C, does not change significantly in response to decreasing the pH from 8 to 4.

**Fig 3:**
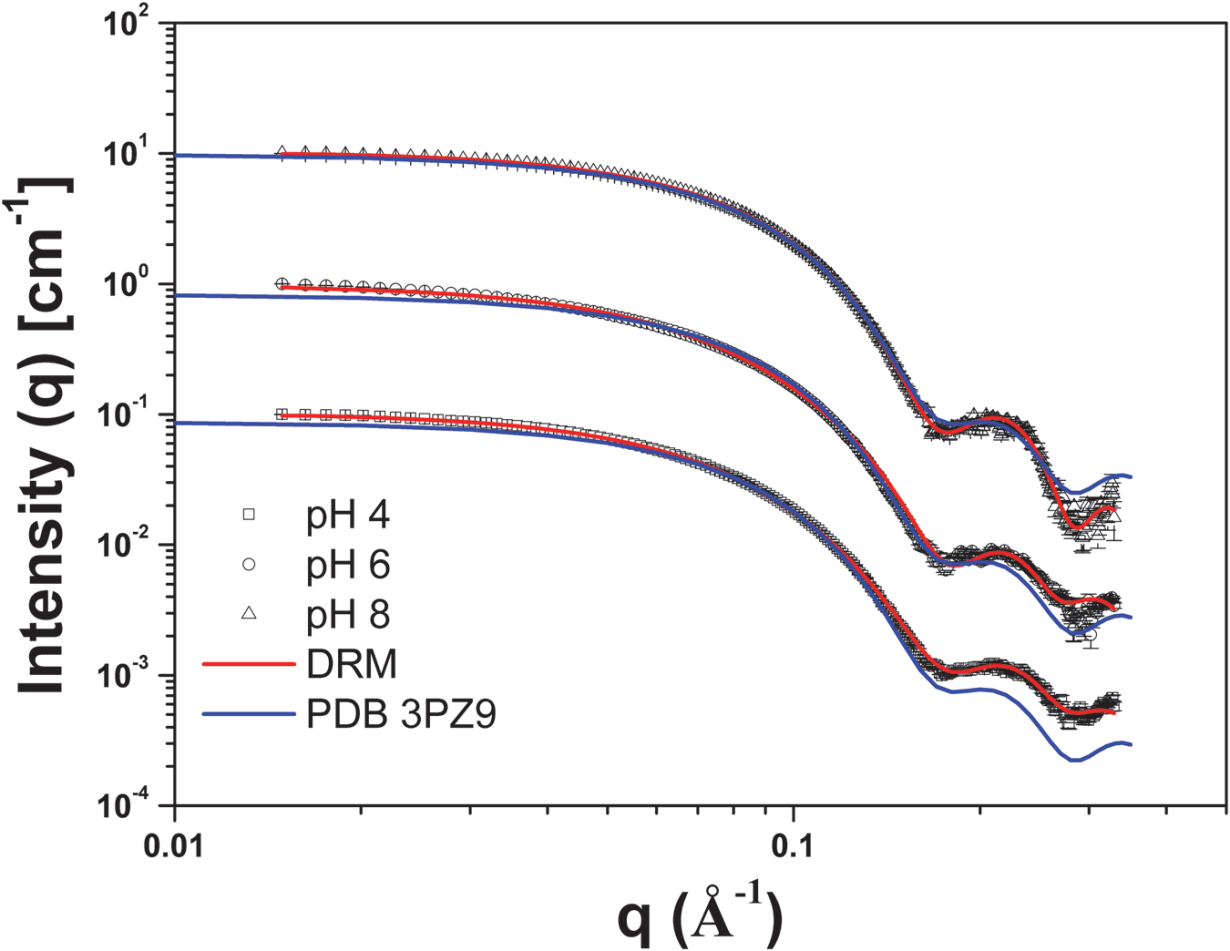
SAXS data collected for TpManGH5 at 20°C and different pH values. Experimental SAXS curves of the TpManGH5 at pH 8 (open black triangles with errors bars), 6 (open black circles with errors bars) and 4 (open black squares with errors bars) superimposed on the computed scattering curves based on the restored low-resolution models (DRM, solid red lines). The solid blue lines represent the computed scattering curve based on the X-ray crystallographic structure of TpManGH5 (PDB 3PZ9). The curves have been offset for clarity.

**Fig 4:**
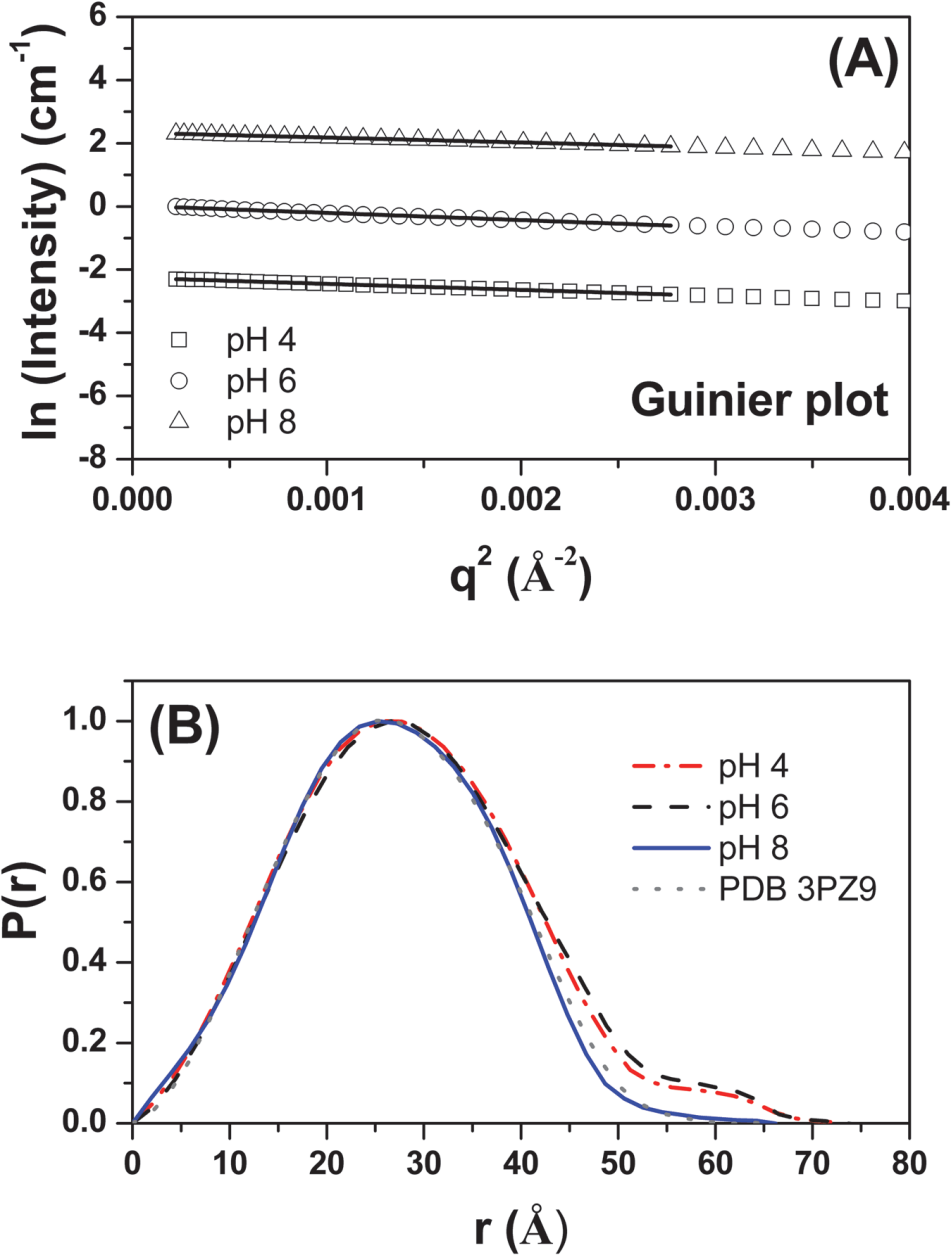
Guinier plots and experimental distance distribution functions. (**A**) Guinier plots (ln *I* versus *q* ^2^) for TpManGH5 at pH 8 (open black triangles), 6 (open black circles) and 4 (open black squares). The fit curves are the solid black lines. The curves have been offset for clarity. (**B**) Experimental *P*(r) of TpManGH5 at pH 8 (solid blue line), 6 (dash black line) and 4 (dash dot red line). The gray dot line represents the theoretical *P*(r) of TpManGH5 calculated from its atomic coordinates (PDB 3PZ9). The curves have been scaled to a maximum height of 1.0 for ready comparison.

SAXS was used to probe the influence of pH on the tertiary structure of the TpManGH5 (Fig. 3). The SAXS intensity profiles for TpManGH5 measured at the three pH values (pH 8, 6, and 4) and the associated Guinier plots are shown in Figs. 3 and 4A, respectively. The Guinier plots are linear, suggesting that the molecules are free of significant intermolecule interference and aggregation. Furthermore, the radii of gyration (*R*_*g*_) determined at different protein concentrations are very close (Table 1, S1 and S2 Figs.). The *R*_*g*_ values determined at pH 8, 6, and 4 were 21.61 ± 0.13 Å, 24.37 ± 0.16 Å, and 23.94 ± 0.12 Å, respectively. The pair distance distribution functions, *P*(r), evaluated by the indirect Fourier transform with GNOM package [18] are shown in Fig. 4B. The profiles have very similar shapes and positions for the main peak, being at 26 Å. The *P*(r) profile at pH 8 has a bell-shape and trail off to a maximum dimension (*D*_*max*_) of 67 ± 2 Å. The *P*(r) profiles at pH 6 and 4 trail off to a *D*_*max*_ of 72 ± 2 Å and 71 ± 2 Å, respectively. There are subtle differences between the profile obtained at pH 8 and acidic medium, with the *P*(r) found at pH 6 and 4 having a clear indication of a small shoulder near 60 Å that does not exist in the profile obtained from the pH 8 data. The *R*_*g*_ values determined for TpManGH5 at pH 8, 6, and 4 using the GNOM package were 21.53 ± 0.02 Å, 24.13 ± 0.01 Å, and 23.35 ± 0.02 Å, respectively. A summary of the main SAXS results described in this study for TpManGH5 is given in Table 2.

**Table 1:** Radius of gyration (*R*_*g*_) calculated with Guinier approximation at different protein concentrations.

C (mg/mL)	*R*_*g*_ (Å) at pH 8	*R*_*g*_ (Å) at pH 6	*R*_*g*_ (Å) at pH 4	*R*_*g*_ (Å) at pH 6, 65°C
1	21.88±0.29	24.16±0.35	23.65±0.22	23.13±0.29
6	21.61±0.13	24.37±0.16	23.94±0.12	23.69±0.13

**Table 2:** General SAXS results from TpManGH5.

Parameters/samples	pH 8 and 20°C	
	**Experimental**	**DRM**
***R***_***g***_ **(Å)**	21.61±0.13 (Guinier)	21.50
	21.53±0.02 (GNOM)	
***D***_***max***_ **(Å)**	67±2	65.18
**Resolution (Å)**	20	-
**χ**	-	2.6

	**pH 6 and 20** ^**o**^ **C**	
	**Experimental**	**DRM**
***R***_***g***_ **(Å)**	24.37±0.16 (Guinier)	24.40
	24.13±0.01 (GNOM)	
***D***_***max***_ **(Å)**	72±2	74.20
**Resolution (Å)**	20	-
**χ**	-	2.9

	**pH 4 and 20** ^**o**^ **C**	
	**Experimental**	**DRM**
***R***_***g***_ **(Å)**	23.94±0.12 (Guinier)	23.30
	23.35±0.02 (GNOM)	
***D***_***max***_ **(Å)**	71±2	72.59
**Resolution (Å)**	20	-
**χ**	-	3.2

	**pH 6 and 65** ^**o**^ **C**	
	**Experimental**	**DRM**
***R***_***g***_ **(Å)**	23.69±0.13 (Guinier)	22.30
	23.32±0.02 (GNOM)	
***D***_***max***_ **(Å)**	71±2	70.36
**Resolution (Å)**	20	-
**χ**	-	3.2

The low-resolution molecular shape of the TpManGH5 was determined from X-ray scattering data with GASBOR package [19] to visualize the shape of the TpManGH5 in solution at different pH values. The resulting consensus envelopes for the pH values studied are available in Supporting Information (S3 Fig.). In each case, ten independent *ab initio* simulations were performed without imposing any symmetry restrictions, and the models described very well the experimental curves. The results, in each case, were compared with each other by the use of the DAMAVER procedure [20] and the most representative model for the whole set is shown in S3 Fig. The experimental X-ray scattering curves superimposed with the theoretical scattering curves from the low-resolution models (DRM, solid red lines) are showed in Fig. 3. The similarity of the low-resolution models is consistent with the *P*(r) analysis. The reconstructed low-resolution model of TpManGH5 at pH 8, restored at 20 Ǻ resolution, showed a shape nearly spherical (globular). However, the reconstructed low-resolution models of TpManGH5 at pH 6 and 4, restored at 20 Ǻ resolution, showed less globular shapes with a region more expanded.

**Fig 5:**
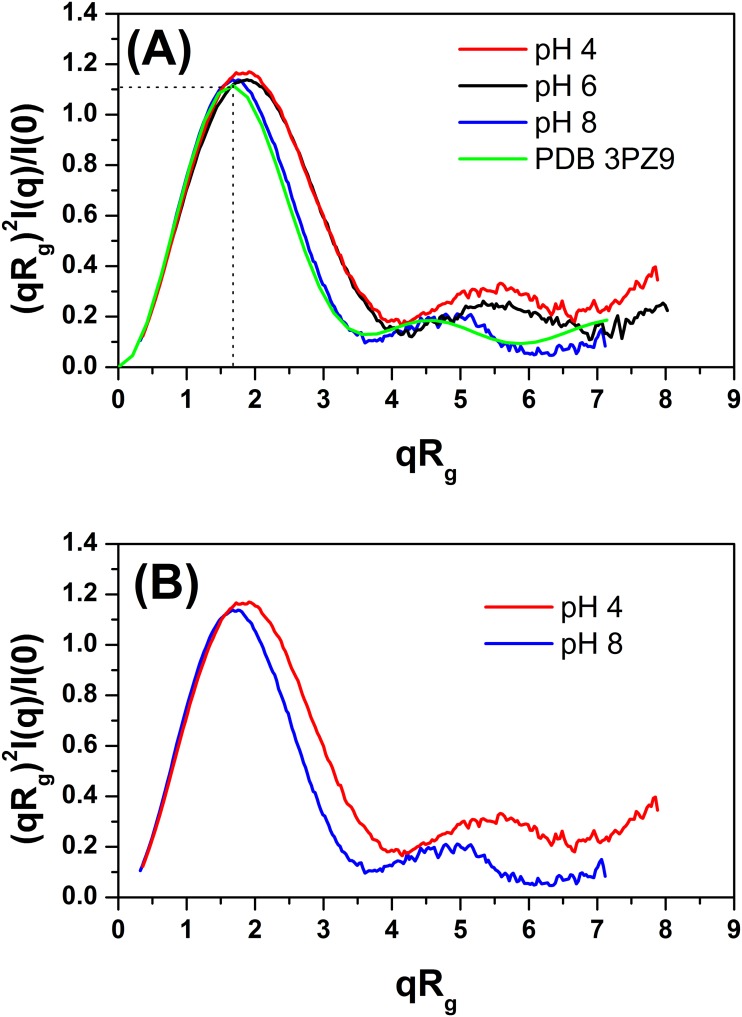
Dimensionless Kratky plots at 20°C and different pH values. (**A**) Dimensionless Kratky plots generated from the SAXS data collected for TpManGH5 at pH 8 (solid blue line), 6 (solid black line) and 4 (solid red line). The solid green line represent the Kratky plot generated from the computed scattering curve based on the X-ray crystallographic structure of TpManGH5 (PDB 3PZ9). (**B**) Dimensionless Kratky plots generated from the SAXS data collected at pH 8 and 4 overlaid to make the differences between the curves.

For a more sensitive analysis of the effects of pH on the global compactness and flexibility of the TpManGH5, SAXS data have been analyzed in terms of the dimensionless Kratky plot [(*qR*_*g*_)^2^I(*q*)/I(0) vs *qR*_*g*_] [21,22]. Kratky plots generated from the SAXS data are shown in Fig. 5A and S4 Fig. As can be seen in Fig. 5A, the curve for TpManGH5 at pH 8 shows a well-defined maximum (*qR*_*g*_ < 3) and decay close to zero at higher *qR*_*g*_ values. Furthermore, the curve at pH 8 shows a maximum value of 1.1 for $qR_{g}=\sqrt{3}$. This behavior is consistent with a nearly globular protein in solution [22]. Thus, TpManGH5 at pH 8 is a prototypical scattering particle with a nearly spherical tightly packed core displaying negligible flexibility in solution [21,22]. The curve for TpManGH5 at pH 6 shows a well-defined maximum and presents a subtle elevated baseline at higher *qR*_*g*_ values (*qR*_*g*_ > 3) when compared to the same enzyme at pH 8, suggesting that the molecule may exhibit some degree of flexibility at pH 6. In contrast, the curve for TpManGH5 at pH 4 does not decay close to zero at higher *qR*_*g*_ values, presenting a significant elevated baseline. This difference at high *qR*_*g*_ is highlighted in Fig. 5B, where the pH 8 and 4 data are compared. This behavior clearly indicates the presence of increased flexibility in the structure at pH 4 when compared to the same enzyme at pH 8 [21,22]. Besides, at acidic pH values, the peaks shifts right with maxima slightly greater than 1.1, indicating that TpManGH5 presents less globular shape [22]. Increased flexibility at pH 4.2 was also observed in the catalytic core domain of the enzyme cellobiohydrolase I from *Trichoderma reesei* [23]. Furthermore, the variations in the flexibility under the different pH scenarios can also be evaluated using the reference curve PDB 3PZ9 in the Fig. 5A (green solid line). While the kinetic behavior to pH 8 is very similar to the crystallographic structure, i.e. presenting small fluctuations around the atomic positions, the other curves deviate from the reference with the flexibility increasing with the acidity.

### Influence of pH on the Number of Uncompensated Charges of the TpManGH5

**Fig 6:**
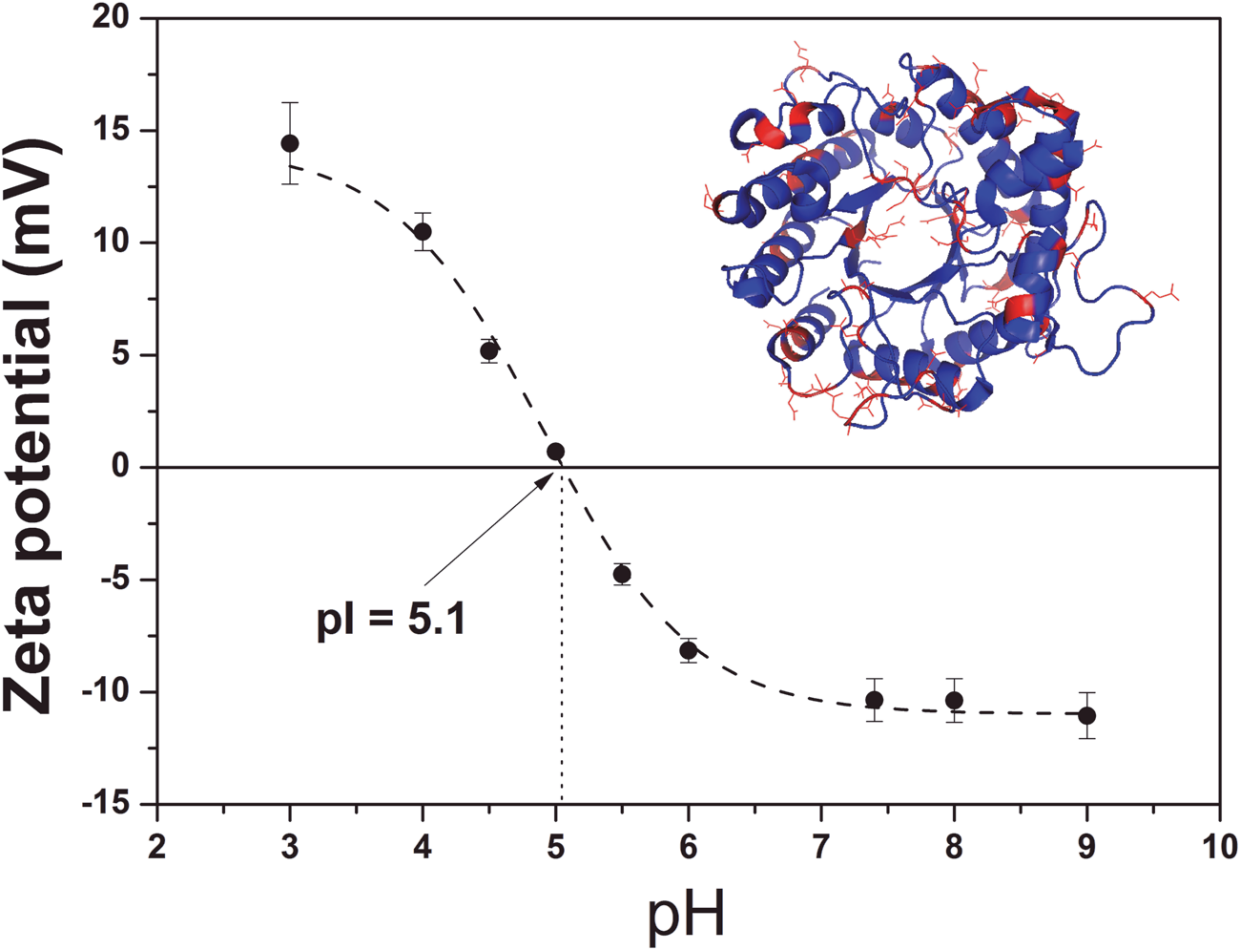
Zeta-potential. Zeta-potential of TpManGH5 as a function of pH. The symbols (black filled circles with errors bars) correspond to the experimental data, and the dash line is just a guide for the eye. *Inset*: a schematic representation of TpManGH5 with the ionizable residues showed in red.

The zeta-potential is an important parameter for understanding electrostatic colloidal dispersion stability. It is a measurement of the charge that a molecule acquires in a particular environment. It depends on the pH, ionic strength, and concentration of a particular component [24,25]. In this work, no attempt was made to study the effect of salt concentration, which might be an important factor in future studies. In order to better understand the influence of pH on the number of uncompensated charges, the zeta-potential of the TpManGH5 was determined as a function of pH. As can be seen in Fig. 6, the zeta-potential of the TpManGH5 decreased from 14.4 ± 1.8 mV at pH 3 to -11.1 ± 0.9 mV at pH 9. The zeta-potential value was close to zero at pH 5.1 ± 0.2 for TpManGH5 (Fig. 6). Our data, using this methodology, suggest that the value determined is consistent with the theoretical pI value (pI = 5.0) calculated from the amino acid sequence [26]. At a pH near the isoelectric point TpManGH5 is unstable at concentrations greater than 1 mg/mL and tends to aggregate. The concentration used in our assays was fixed at 1 mg/mL, which was found to be adequate to obtain reproducible data.

The electrophoretic mobility (*μ*_*e*_) measurements can give the average translation velocity of the protein, *V*, under a given electric field *E*. Hence, the electrophoretic mobility is defined as: $\mu_{e}=\;\frac{V}{E}$. Knowledge of the electrophoretic mobility enables one to calculate the average number of charges per molecule (*N*_*c*_) from the Lorenz-Stokes relationship [24,25]: $N_{c}=\frac{6\pi \eta }{e}R_{S}\mu_{e}$, where *μ*_*e*_ is expressed in μm.cm.s^-1^.V^-1^ and the value in the denominator corresponds to the elementary charge (*e* = 1.602 x 10^-19^ C). The experimental values of the electrophoretic mobility and the net number of uncompensated charges per molecule are shown in Table 3. For pH < 5.1, TpManGH5 acquire positive charges that increase with decreasing of pH. However, for pH > pI, TpManGH5 acquire negative charge. The *N*_*C*_ values determined at pH 8, 6, and 4 were -2.37 ± 0.23, -1.84 ± 0.32, and 2.48 ± 0.21, respectively.

**Table 3:** Electrophoretic mobility (***μ***_***e***_) and the number of uncompensated charges (***N***_***c***_) for TpManGH5 at different pH values.

TpManGH5			
pH	*μ*_*c*_ (μm.cm/Vs)	*R*_*S*_ (Ǻ)	*N*_*c*_
3.0	1.13±0.14	31±3	3.67±0.45
4.0	0.82±0.07	29±2	2.48±0.21
4.5	0.42±0.06	28±2	1.23±0.18
5.0	0.05±0.01	29±2	0.15±0.03
5.5	-0.37±0.04	28±2	-1.09±0.11
6.0	-0.63±0.11	28±2	-1.84±0.32
7.4	-0.81±0.08	28±2	-2.38±0.23
8.0	-0.81±0.08	28±2	-2.37±0.23
9.0	-0.93±0.15	29±2	-2.82±0.46

### Influence of Temperature on the Structural Stability and Global Compactness of the TpManGH5

Influence of temperature on the structural stability of the recombinant TpManGH5 was studied using far-UV CD spectroscopy, dynamic light scattering (DLS) and SAXS. The thermal stability of TpManGH5 was characterized at different pH values following changes in the CD ellipticity at 220 nm as a function of temperature (Fig. 2B and S5 Fig.). At temperatures lower than 75°C, at all pH values, the ellipticities at 220 nm are constant, suggesting a stable regular secondary structure (Fig. 2B). However, a progressively decrease in the ellipticities were observed, at all pH values, when the temperature was increased above 80°C (Fig. 2B). These alterations indicated that TpManGH5 loses regular secondary structure at temperatures higher than 80°C with a complete unfolding at 95°C. The protein melting point (*T*_*M*_) is defined as the temperature at which the protein denatures. The *T*_*M*_ values for TpManGH5 at pH 8, 6 and 4 were calculated and resulted in 83 ± 1°C, 88 ± 1°C, and 80 ± 1°C, respectively (Fig. 2B). Thus, the better thermostability is reached at pH 6. At all pH values, the processes were essentially irreversible, in the conditions described here, as can be deduced from the unchanged values of the ellipticity at 220 nm obtained upon cooling the samples back to 20°C after denaturation at 95°C (data not shown).

**Fig 7:**
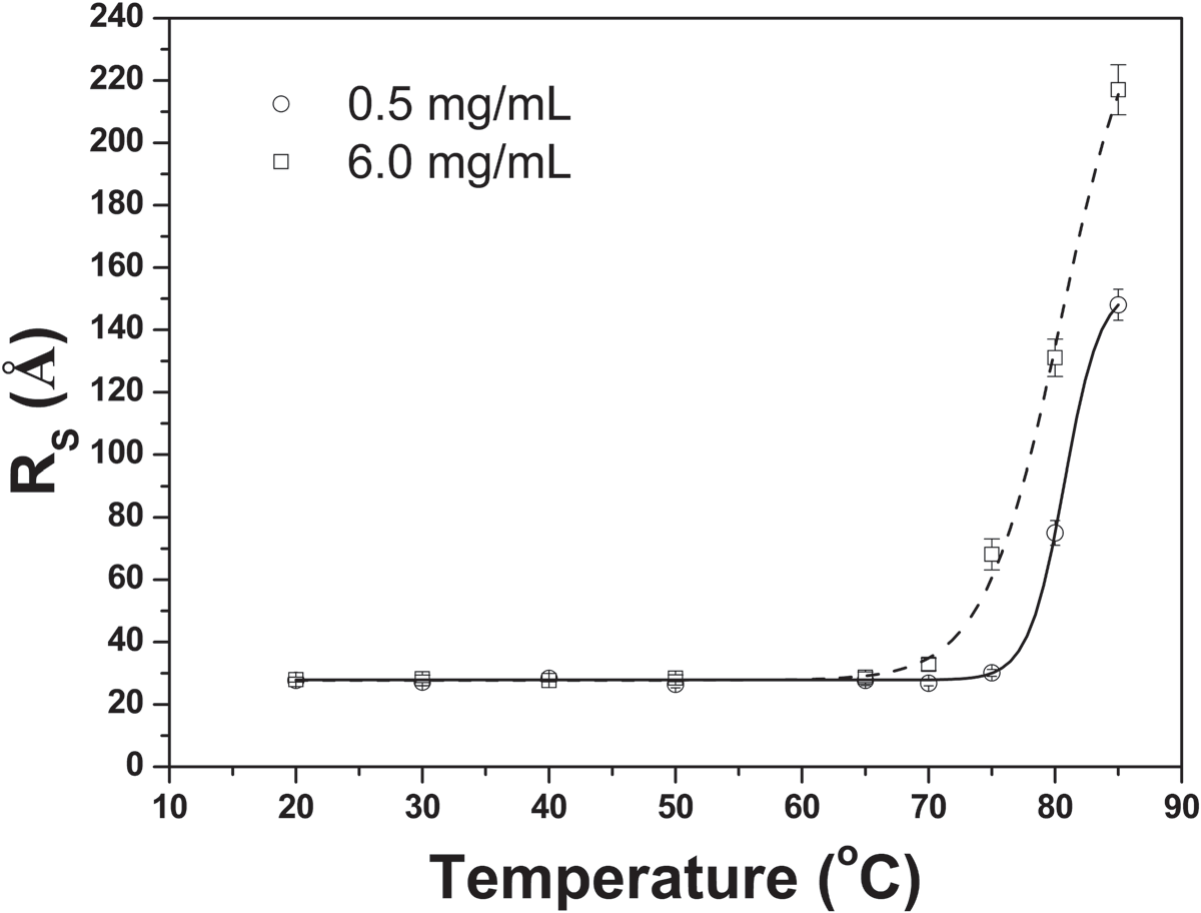
Characterization of TpManGH5 by DLS at pH 6. The hydrodynamic radius (*R*_*S*_) of TpManGH5 at different concentrations (0.5 and 6 mg/mL) monitored as a function of temperature.

DLS has long been used as a protein characterization tool [24,27]. This method is ideally suited for monitoring the stability of the protein structure to denaturing or aggregation conditions. DLS size distribution profiles for TpManGH5 are available in Supporting Information (S6 Fig.). As can be seen in Fig. 7, the hydrodynamic radii (*R*_*S*_) of the TpManGH5 exhibited minimal temperature dependence over the range 20 to 75°C at pH 6 and 0.5 mg/mL, suggesting a stable tertiary structure. After incubation at 75°C there was no evidence of enzyme precipitation and the solution remained transparent. The average *R*_*S*_ determined for TpManGH5 by DLS method was 28 ± 2 Ǻ, which corresponds to a molecular mass of 38 ± 5 kDa, entirely consistent with the expected molecular mass of 42 kDa (S7 Fig.). However, when TpManGH5 at pH 6 and 0.5 mg/mL was incubated at temperature values above 75°C the *R*_*S*_ increased significantly, suggesting a tendency to form amorphous aggregates (Fig. 7). After incubation at 85°C and 0.5 mg/mL the initially clear TpManGH5 solution was very turbid and precipitate had formed. For TpManGH5 at pH 6 and 6 mg/mL, the *R*_*S*_ was constant at temperatures lower than 65°C, suggesting a stable tertiary structure. At 70°C and higher, the *R*_*S*_ increased with the increase in temperature, indicating the presence of denatured aggregates. Again, after incubation at 75°C and 6 mg/mL the initially clear TpManGH5 solution was very turbid and precipitate had formed.

**Fig 8:**
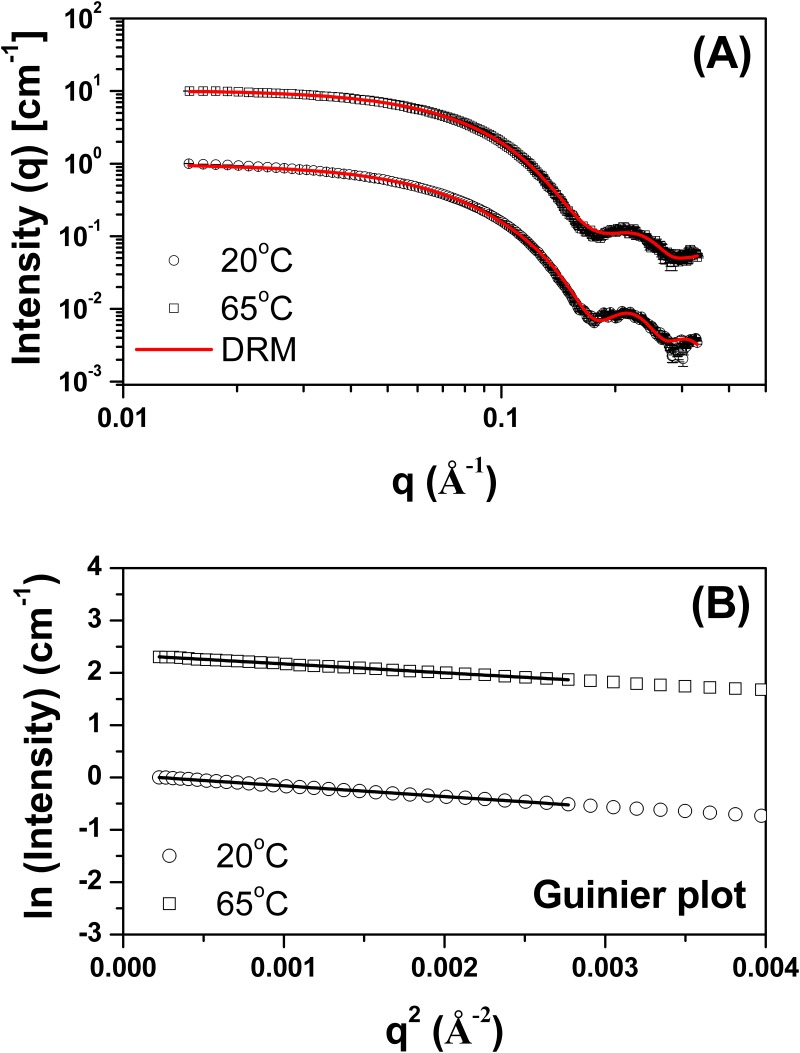
SAXS data collected for TpManGH5 at pH 6 and different temperatures. (**A**) Experimental SAXS curves of the TpManGH5 at 20°C (open black circles with errors bars) and 65°C (open black squares with errors bars) superimposed on the computed scattering curves based on the restored low-resolution models (DRM, solid red lines). The curves have been offset for clarity. (**B**) Guinier plots (ln *I* versus *q* ^2^) for TpManGH5 at 20°C (open black circles) and 65°C (open black squares). The fit curves are the solid black lines. The curves have been offset for clarity.

**Fig 9:**
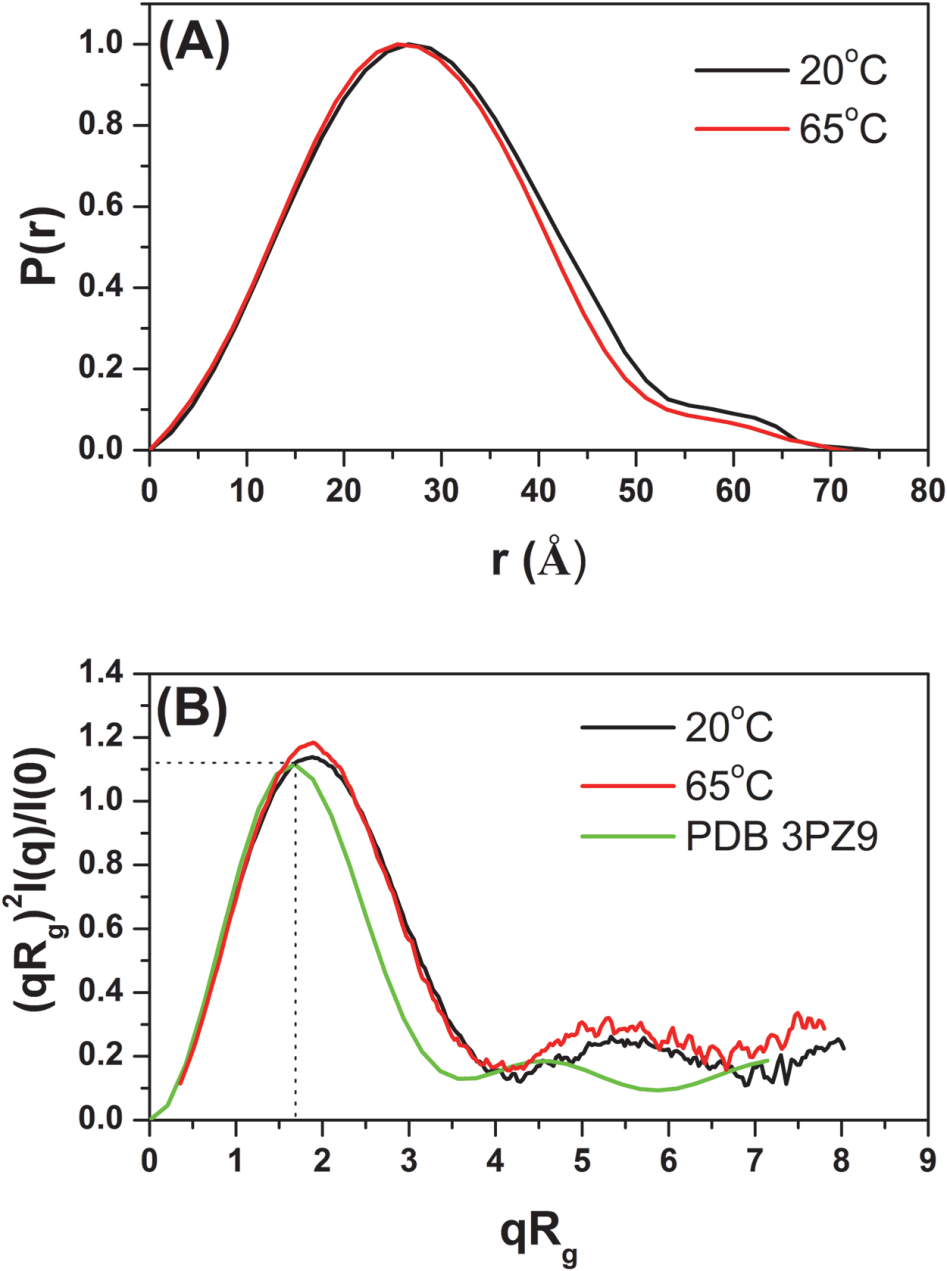
Experimental distance distribution functions and dimensionless Kratky plots at different temperatures. (**A**) Experimental *P*(r) of TpManGH5 at 20°C (solid black line) and 65°C (solid red line). The curves have been scaled to a maximum height of 1.0 for ready comparison. (**B**) Dimensionless Kratky plots generated from the SAXS data collected for TpManGH5 at 20°C (solid black line) and 65°C (solid red line). The solid green line represent the Kratky plot generated from the computed scattering curve based on the X-ray crystallographic structure of TpManGH5 (PDB 3PZ9).

The SAXS intensity profiles for TpManGH5 measured at 20 and 65°C and the associated Guinier plots are shown in Fig. 8. Scattering curves obtained at 70°C and higher concentration showed nonnegligible interference effects, and they were not used for analyzes (data not shown). Like previously, the Guinier plots are linear, suggesting that the molecules are free of significant intermolecule interference and aggregation (Table 1 and S1 Fig.). The *R*_*g*_ evaluated with the Guinier approximation at 20 and 65°C were 24.37 ± 0.16 Å and 23.69 ± 0.13 Å, respectively. Therefore, the *R*_*g*_ does not change significantly when the temperature increases from 20 to 65°C. The *P*(r) evaluated by the indirect Fourier transform with GNOM package [18] are shown in Fig. 9. The *P*(r) profiles at 20 and 65°C have very similar shapes and trail off to a *D*_*max*_ of 72 ± 2 Å and 71 ± 2 Å, respectively. The *R*_*g*_ values determined for TpManGH5 at 20 and 65°C, using the GNOM package, were 24.13 ± 0.01 Å and 23.32 ± 0.02 Å, respectively (Table 2). The resulting consensus envelopes for 20 and 65°C are shown in Supporting Information (S8 Fig.). The low-resolution models obtained are consistent with the *P*(r) analysis. The experimental X-ray scattering curves superimposed with the theoretical scattering curves from the low-resolution models (DRM, solid red lines) are shown in Fig. 8A. Similarly at 20°C and pH 6, the reconstructed low-resolution model of the TpManGH5 at 65°C and pH 6, restored at 20 Ǻ resolution, showed less globular shape and a region slightly more expanded. Dimensionless Kratky plots generated from the SAXS data are shown in Fig. 9B. The curve for TpManGH5 at 65°C and pH 6 shows a well-defined maximum and presents a subtle increase of the baseline when compared to the same enzyme at 20°C. This result suggests that TpManGH5 is slightly more flexible in solution at 65°C when compared to the same enzyme at 20°C.

### SAXS Characterization Through Molecular Dynamics Simulations of Flexible Models

**Fig 10:**
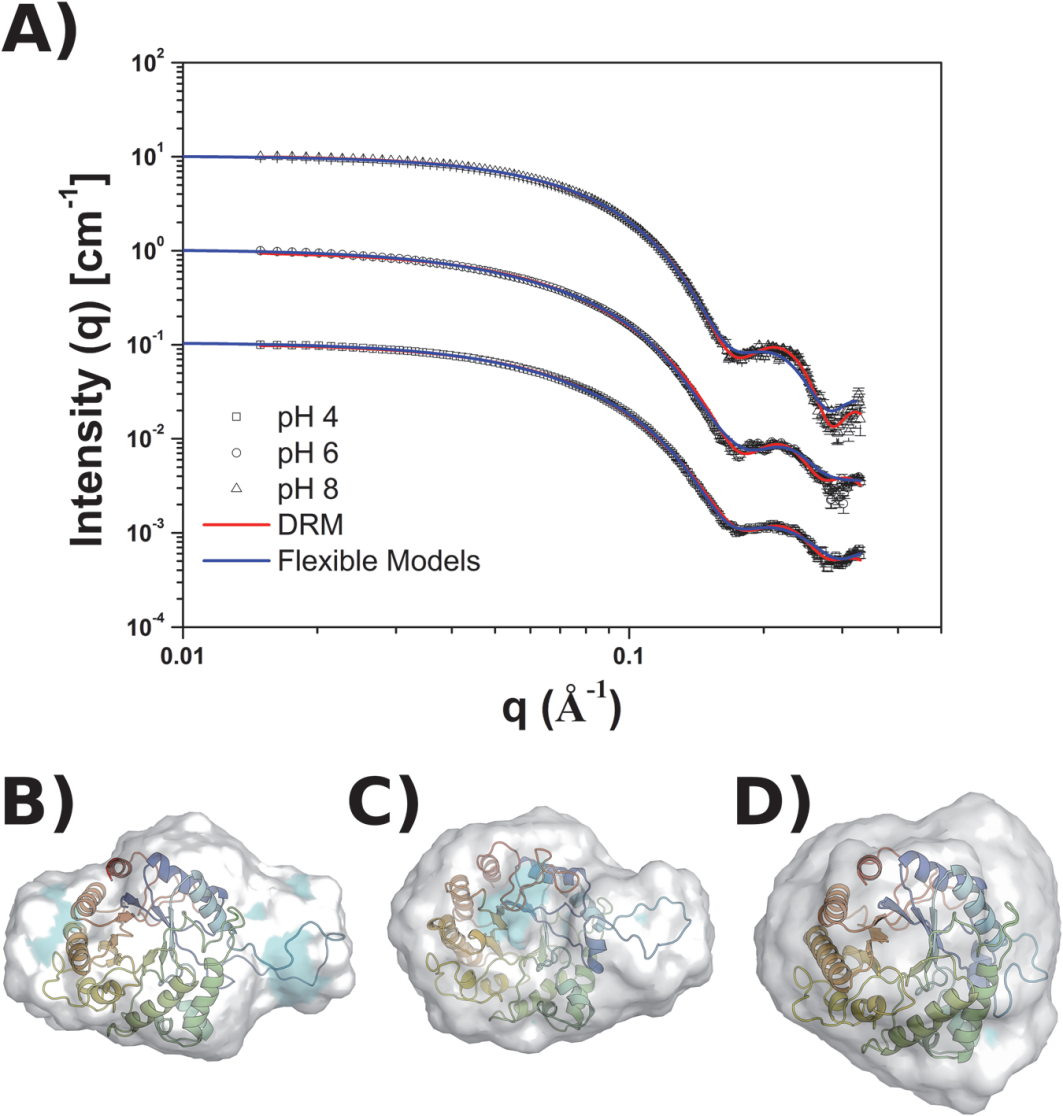
Molecular dynamics simulations. **(A)** Experimental and theoretical SAXS curves of the TpManGH5 at 20°C for the different pH scenarios. The theoretical curves were obtained from independent simulations and calculated using CRYSOL package. It is showed only the conformation with the lowest χ^2^ for each pH selected. The theoretical curves for flexible models (solid blue lines) and DRM (solid red lines) are overlapped in the experimental curves to the pH equals 8 (triangles), 6 (circles) and 4 (squares). The cartoon representations of the conformation with the best agreement with the experimental data are also presented. They show the models superimposed into the *ab initio* DRM low-resolution envelopes calculated using GASBOR to the pH 4 (**B**), 6 (**C**) and 8 (**D**). These Figures illustrate the variation in the elongation of the loop (residues Y88 to A105), indicating the region where conformational changes occurs and its flexibility for the different pHs. These results, combined with biochemical evaluation, suggest that the dynamics of the loop associated to the protonation state may define a balance for the optimal enzymatic activity. It also suggested that fluctuations in the loop may play an important role for the hydrolysis. Even, it is possible verify that the remaining part of the protein, i.e. the core of the protein, is not affected by the conformational changes of the loop. The blue spots in the low-resolution molecular envelopes indicate the shell hydration and support the hypothesis of the mechanical movement of the loop facilitate access to the water molecule, necessary during the hydrolysis.

Previous SAXS analysis indicated that at acidic pH conditions TpManGH5 shows a less globular shape, probably due to a loop region (residues Y88 to A105) slightly more expanded and flexible in solution. To investigate in more detail this hypothesis, it is necessary a computational tool able to generate physically possible deformations, that means, conformations limited by protein geometrical and energetic features. Molecular dynamics simulations of Structure Based Models (SBM) provide an efficient way to search the conformational space, allowing a more detailed characterization of the TpManGH5 under the different scenarios of pH [28–30]. The present approach compares conformations obtained from simulations of all-atoms models with SAXS experimental data. By employing different contact maps, i.e. constructions relaxing target regions, it is possible to generate a representative conformation of the enzyme in solution based on the comparison with the SAXS curves for each pH value. The simulations were performed using 15 different contact maps, composed by the combinations of 4 regions (loop 1: Y88-A105; loop 2: L195-S206; loop 3: A231-K245 and loop 4: L352-G372) selected using the Frustratometer web server [31] (S9 Fig.). The interactions between these regions and the rest of the protein were removed providing 14 different energetic potential models plus the original contact map without modifications. Theoretical scattering curves were calculated for each conformation obtained from the different simulations and the agreement between the theoretical and experimental data were evaluated using the parameter χ^2^, calculated using CRYSOL package [32]. The theoretical and experimental curves are shown in Fig. 10. Furthermore, a cartoon representation inside the low-resolution models obtained using GASBOR package is shown in the same Figure. The simulations did not provide any better fit to pH 8 than crystallographic structure (PDB 3PZ9), whose χ^2^ and *R*_*g*_ are equal to 3.2 and 21.50 Å, respectively (Fig. 10). The curves at acidic pH conditions showed a good fit with the simulations performed using the contact map where only the Lennard-Jones interactions between the loop composed by the residues Y88 to A105 (index based on the crystallographic structure) were removed. The conformations with the best adjust provided a χ^2^ equals 2.9 and *R*_*g*_ of 22.20 Å for the pH 6 and a χ^2^ equals 2.5 and *R*_*g*_ of 22.30 Å for the pH 4 (Fig. 10). These results indicate that only this specific region is responsible for the change in the SAXS intensity signal, being a consequence of the conformation change of the loop. Also, it was possible to identify that conformational changes did not change the core of the TpManGH5, which means that only the loop region presents high degree of fluctuations, not changing the stability of the protein due to the loop movement. The S1 Table in the Supporting Information presents the lower χ^2^ found for each one of the 14 model constructions.

A possible explanation of effects caused by the conformational changes was studied identifying and evaluating the binding pocket volume through the DogSiteScorer web server [33] (Table 4). The representative conformation obtained from the structural characterization by flexible models simulations to pH 6 presented an increase around 11% in the binding pocket volume when compared to the crystallographic structure. Thus, as consequence of the conformational change the active site become more accessible to substrate than in the conformation found to pH 8. Unlikely, the binding pocket volume of the representative conformation to pH 4 shows an increase around 74% when compared to the pH 8 conformation. Certainly, the active site also become more accessible to the substrate, however, the residues of the binding pocket are slightly spread. Globally, the structure presents minor modifications in the core, but slightly higher inter-residues distances in active site. As a consequence, the interactions between enzyme and substrate cannot be properly satisfied as to pH 6, resulting in efficiency losses as showed in the Fig. 1A. The S2 Table in the Supporting Information presents the inter-residue distances in the active site obtained for pH 8, 6 and 4.

**Table 4:** Pocket identification and calculation.

pH	Volume (Å^3^)	Surface (Å^2^)
4	746.7	1052.8
6	477.3	722.4
8	429.6	474.4

The pocket surface and volume were calculated using the Web Server DogSiteScorer (dogsite.zbh.uni-hamburg.de) [33] to the three-dimensional conformations obtained from molecular dynamics simulations with the lowest χ^2^ against the experimental SAXS results to pH 4, 6 and 8. The analysis shows an increase of the volume and surface with acidity increase. When the pH changes from 8 to 6 the pocket volume increases around 11%, becoming more accessible to ligands. However, when the pH goes from 8 to 4 the volume of the pocket increases around 74%. This variation may become the pocket more accessible, but the distance between the residues of the active site are less suitable for the hydrolysis.

The pKa was also estimated using PropKa 3.0 through the PDB2PQR web server [34,35] to evaluate the consequences of charge distribution due to pH variation (Table 5 and S3 Table). The protonation state of the residues were calculated using the Henderson-Hasselbalch equation (log([A^-^]/[HA]) = pH-pKa) where a ratio [A^-^]/[HA] lower than 1 indicates a protonated residue or a deprotonated residue when higher than 1. The analysis shows the residues H90, E92 and E100 deprotonated to pH 8 and pH 6, but protonated to pH4. For this reason, the loop 1 becomes more extended with the decrease of the pH. This conformational change, due to the pH variation, also affects the residues of the active site. Thus, the residues E235 and H283 are protonated only to pH 4 and pH 6, while E198 is deprotonated for all the cases, but with a higher probability to pH 6 than other cases. It is also important to note the presence of catalytic residues in the loop 2 (E198 and R200) and loop 3 (E235), thus extensions of these regions are not appropriated under the activity perspective. The analysis of the loop 4 is unclear under the viewpoint of pKa, since it indicates the protonation of the residues 357, 363, 369 and 371 with the increase of acidity. The region may suffer variations not captured by the models or analysis. Anyway, the result indicates the importance of the protonation state and conformational changes and it illustrates how this balance may interfere on the enzymatic activity.

**Table 5:** Estimative of the protonation state.

	[A^-^]/[HA]		
Residue	pH 4	pH 6	pH 8
Loop 1			
88	*0*	*0*.*0001*	*0*.*0015*
90	*0*.*0037*	**1.2882**	**331131.1**
92	*0*.*2344*	**11.749**	**549.5406**
100	*0*.*2884*	**22.9087**	**2818.382**
Loop 2			
198	**44.6684**	**123.0269**	**1.5136**
200	*0*	*0*	*0*
201	*0*	*0*	*0*.*0001*
202	*0*.*2754*	**43.6516**	**2570.395**
204	**2.138**	**954.9926**	**4265797**
205	*0*	*0*.*0001*	*0*.*0083*
Loop 3			
234	*0*.*0035*	*1*.*3183*	**13.1826**
235	*0*	*0*	**3.02**
241	*0*	*0*	*0*
242	*0*.*389*	**26.3027**	**2884.032**
245	*0*	*0*	*0*.*0033*
Loop 4			
357	*0*.*1995*	**5.8884**	**51286.14**
360	**5.0119**	**58.8844**	**354813.6**
361	*0*	*0*	*0*
362	**12.5893**	**4786.302**	**1318256**
363	*0*.*2138*	**2.3988**	**9120.109**
364	*0*	*0*	*0*
366	*0*	*0*	*0*.*0028*
367	*0*	*0*	*0*
369	*0*.*4266*	**758.5778**	**24547.11**
370	*0*	*0*	*0*.*0001*
371	*0*.*0219*	*0*.*4266*	**3019.952**
Active site			
198	**44.6684**	**123.0269**	**1.5136**
200	*0*	*0*	*0*
235	*0*	*0*	**3.02**
283	*0*.*0083*	*0*.*5623*	**162.181**

The protonation state of the loops and active site residues were calculated through the values of the pKa provided by PROPKA 3.0. The evaluation was performed using the representative conformation for each scenario on its pH. The relation [A^-^]/[HA] was obtained employing the Henderson-Hasselbalch equation (log([A^-^]/[HA]) = pH-pKa). Values lower and higher than 1 means a residue protonated (*italic*) and deprotonated (**bold**), respectively. The analysis indicates a positive variation of charge in H90, E92 and E100 with the increase of the acidity, explaining the extension of the loop 1. Also, as consequence of the conformational change and pH variation, the residues E198, E235 and H283 show differences to pH 4 and pH 6 when compared to pH 8. The charge variation of these constituents of the active site illustrates the role of balance between the extension of the loop 1 and the protonation state of key residues of the active site.

## Discussion

The biodegradation of mannan-containing polysaccharides involves a concerted attack by several enzymes, including endo-β-1, 4-mannanase. This process has attracted the attention of researchers because it represents a key step for several industrial applications including delignification of kraft pulps [14,15], food processing [8,16] and production of second-generation biofuels [6,8,16]. Furthermore, for industrial applications, β-mannanases stable and functional at high temperatures offer substantial techno-economical advantages. A recent study reported that the deletion of the linker plus TpManCBM27 from TpMan has no statistically relevant effect on the catalytic efficiency upon both mannan and glucomannan substrates [10]. For both substrates, the truncated catalytic domain showed higher *V*_*max*_. However, the deletion of the linker plus TpManCBM27 reduced the thermal stability of the TpManGH5 [10]. Despite reduction of the thermostability, TpManGH5 reaches its optimum activity at 75°C and pH 6 (Fig. 1B), applicable condition for several biotechnological processes.

The values of the radii of gyration of the TpManGH5 at alkaline pH (pH 8 and 20°C) calculated from both the Guinier region of the SAXS scattering curve (*R*_*g*_ = 21.61 ± 0.13 Å) and that obtained with GNOM package (*R*_*g*_ = 21.53 ± 0.02 Å) are in excellent agreement (Fig. 4 and Table 2). The reconstructed low-resolution model of the TpManGH5 at pH 8 shows a nearly globular molecule (S3 Fig.). Furthermore, the *P*(r) of the TpManGH5 has a bell-shape (Fig. 4B, solid blue line), which is characteristic of globular proteins. Superposition of the crystallographic structure of the TpManGH5 and the low-resolution model shows an excellent agreement (S3 Fig.). This is supported by the comparison of the experimental X-ray scattering curve and the computed theoretical scattering curve based on the crystallographic structure of TpManGH5 (Fig. 3, solid blue line). The fit is excellent, which indicates that the size and shape of TpManGH5 in solution at pH 8 are nearly identical to what were seen for the X-ray crystal structure (*R*_*g*_ = 20.87 Å and *D*_*max*_ = 65.74 Å) [10].

At acidic pH (pH 6 and 4, 20°C), the values of the *R*_*g*_ of the TpManGH5 calculated from the Guinier regions are very close to the values obtained from the integral analysis of the scattering curves using the method implemented in the GNOM package (Table 2). TpManGH5 experiences an increase in *R*_*g*_ at acidic conditions suggesting expansion of the molecule. In both cases, the reconstructed low-resolution models showed less globular shapes with a more expanded region (S3 Fig.). Superposition of the crystallographic structure of the TpManGH5 and the low-resolution models, at pH 6 and 4, showed a reasonable agreement. However, there is a loop region (residues Y88 to A105) on the atomic resolution model that seems to be more expanded on the low-resolution models. This is also supported by comparison of the experimental X-ray scattering curves and the computed theoretical scattering curve based on the crystallographic structure of the TpManGH5 (Fig. 3, solid blue line). The fits are not perfect, which indicates that there are small differences between the crystal structure and TpManGH5 in solution at pH 6 and 4, probably because the loop region above mentioned is slightly more expand and flexible in solution. The values of the radii of gyration of TpManGH5 at 65°C (pH 6) calculated from both the Guinier region (*R*_*g*_ = 23.69 ± 0.13 Å) and that obtained with GNOM package (*R*_*g*_ = 23.32 ± 0.02 Å) are in excellent agreement (Fig. 8 and Table 2). Similarly at 20°C, the reconstructed low-resolution model at 65°C showed less globular shape with a more expanded region (S8 Fig.).

The SAXS studies presented here demonstrated that TpManGH5 becomes gradually more flexible in response to decreasing the pH from 8 to 4. The determination of the net number of uncompensated charges per molecule on the TpManGH5 shed light on the origin of the pH-dependent change. As can be seen in Fig. 6, the results show little change in the *N*_*C*_ of the TpManGH5 at pH 8 (-10.37 mV, *N*_*C*_ = -2.37 ± 0.23) and 6 (-8.15 mV, *N*_*C*_ = -1.84 ± 0.32), in agreement with the subtle increase in the flexibility observed in the Kratky plot at pH 6 (Fig. 5A, solid black line). However, an abrupt and strong change in the *N*_*C*_ of the TpManGH5 takes place between pH 6 and 4, with the *N*_*C*_ changing from *N*_*C*_ = -1.84 ± 0.32 (-8.18 mV) to 2.48 ± 0.21 (10.49 mV). This strong change is likely to be responsible for the increased flexibility seen in the dimensionless Kratky plot at pH 4 (Fig. 5, solid red line). The residues affected are shown in red in the Fig. 6 (inset) and are distributed throughout the TpManGH5. Most of the ionizable residues lie on surface of the TpManGH5. Furthermore, the amino acids E92 and E100 are present in the loop region, on the atomic resolution model, that seems to be more expanded on the low-resolution model at pH 6 and 4. Finally, the results indicated that TpManGH5 is slightly more flexible in solution at 65°C when compared to the same enzyme at 20°C (Fig. 9B). Taken together, the results presented here indicated that TpManGH5 becomes appropriately flexible at pH 6 and high temperature (65°C), conditions close to the optimal activity, while retaining its secondary and tertiary structures.

The structure based models employed do not take into account the charges of amino acids/residue groups, however, by using variations in the contact map it is possible to perform a reasonable conformational searching selecting good representative conformations for each pH evaluated and identifying interesting regions. A recent study of Bu et. al. [36] indicates other enzymes where the activity is directly related by the balance between protonation and conformational changes. It allows to classify enzymatic activity exclusively by the protonation of the binding site or by the combination of the mechanical and charge redistribution as consequence of pH variation. In this study, several indicatives were obtained to understand that the optimal activity is dependent of the mentioned balance, but not an exclusive consequence of the charges redistribution in TpManGH5. The identification of this region can be considered the starting point for a rational design of the TpManGH5 based on the mobility of the loop where the conformational changes occurs, for instance enabling the enzyme to work on a more suitable range of pH, enhancing its biotechnological potential. In addition, it must be highlighted that TpManGH5 is hyperthermophilic, so a protein engineering process may not affect drastically its regime of thermal fluctuations, empowering the enzyme interest for industrial processes.

The results from molecular dynamics simulations and active site volume (Table 4) also suggest how the conformational change of the loop 1 may facilitate polysaccharide and enzyme interaction. The solvation shell obtained from molecular envelopes superimposed with the representative three-dimensional conformations shows, for all pH evaluated, water molecules around the active site and loop 1, as presented in Fig. 10. Considering the importance of waters molecules close of the catalytic site for the hydrolysis, it is possible take into account the role that conformational changes plays to facilitate the accessibility of water in the catalytic site, and increasing the relative activity as consequence. The results also corroborates with the number of uncompensated charges described above (Fig. 6).

## Conclusions

We demonstrated that TpManGH5 at pH 8 is a molecule with a nearly spherical tightly packed core displaying negligible flexibility in solution, and with size and shape very similar to crystal structure. However, TpManGH5 experiences an increase in radius of gyration at acidic conditions suggesting expansion of the molecule. Furthermore, at acidic pH values TpManGH5 showed less globular shape, probably due to a loop region slightly more expanded and flexible in solution. In addition, molecular dynamics simulations indicated that conformational changes caused by pH variation did not change the core of the TpManGH5, which means that only the above mentioned loop region presents high degree of fluctuations. The results also suggested that conformational changes of the loop region may facilitate polysaccharide and enzyme interaction. Finally, at pH 6 the results indicated that TpManGH5 is slightly more flexible at 65°C when compared to the same enzyme at 20°C. The biophysical characterization presented here is well correlated with the enzymatic activity and provides new insight into the structural basis for the temperature and pH-dependent activity of the TpManGH5. Also, the data suggest a loop region that provides a starting point for a rational design of biotechnological desired features.

## Materials and Methods

### Materials

Nickel-nitrilotriacetic acid resin (Ni-NTA), imidazole, kanamycin, LB medium, isopropyl-β-D-thiogalactopyranoside (IPTG) and locust bean gum were purchased from Sigma-Aldrich. All chemicals and reagents used in this study were of the highest purity analytical grade.

### Expression and Purification of Recombinant TpManGH5

The expression and purification of the isolated catalytic core domain (TpManGH5) were carried out as described previously with slight modifications [10], and its purity was verified by 15% SDS-PAGE. The resulting TpManGH5 was exhaustively dialyzed in 20 mM acetate-borate-phosphate buffer adjusted at pH 7.5 for elimination of imidazole and NaCl. TpManGH5 concentration was determined by UV absorbance at 280 nm using a theoretical extinction coefficient based on the amino acid composition. The theoretical coefficient employed was ε_280nm_ = 108,875 M^-1^cm^-1^. The final TpManGH5 product was then frozen and stored at -80°C and was melted on ice before use.

### Enzymatic Activity Measurements

Mannan endo-1, 4-β-mannosidase activity was determined using as substrate 1% against locust bean gum (galactomannan) dissolved in a buffer. Activity of TpManGH5 was measured at 75°C and pH values from 4 to 10. Furthermore, activity of TpManGH5 was measuredat pH 6 and temperature range between 20 and 85°C. TpManGH5 was initially incubated during 30 minutes in each temperature and subsequently the remaining enzymatic activity was determined. It was used 20 mM acetate-borate-phosphate buffer adjusted to the different pH values. The reaction in each experiment was performed by mixing 20 μL of the diluted enzyme (1.5 μM) with 80 μL of galactomannan for 10 min. The reaction was stopped by the addition of DNS reagent followed by boiling the samples at 100°C water bath for 5 min [37]. One unit of mannan endo-1, 4-β-mannosidase activity was defined as the amount of enzyme needed to release 1 μmol of mannose equivalent per minute. All experiments were done in triplicate, and average values are reported. The relative activity is given in function of the maximum value of activity normalized in the range evaluated.

### Far-UV Circular Dichroism (CD) Spectroscopy

Far-UV CD spectra were collected using a Jasco J-815 spectropolarimeter equipped with a temperature control device. TpManGH5 concentration was 5 μM in 20 mM acetate-borate-phosphate buffer adjusted to the different pH values. All data were collected at 20°C and 75°C using 0.1 cm quartz cuvette and the spectra were recorded over the wavelength range from 195 to 260 nm. Eight accumulations were averaged to form the CD spectra, taken using a scanning speed of 100 nm min^-1^, a spectral bandwidth of 1 nm, and a response time of 0.5 s, and obtained on degree scale. The buffer contribution was subtracted in each of the experiments. Spectra were transformed to molar ellipticity [θ] using the mean weight residue and concentration prior to the secondary-structure analysis. Thermal unfolding of TpManGH5 (5 μM) was characterized by measuring the ellipticity changes at 220 nm at different pH values induced by a temperature increase from 20°C to 95°C at a heating rate of 10°C/h. The fraction of denatured protein (α) is calculated from the relationship: α = (θ_n_−θ_obs_)/ (θ_n_−θ_d_) and α + β = 1, in which θ_obs_ is the ellipticity of the sample at a particular condition; θ_d_ and θ_n_ are the values of ellipticity characteristic of the denatured and native states.

### Dynamic Light Scattering (DLS)

The size characteristics of the purified TpManGH5 samples were examined by means of the Nano-ZS dynamic light scattering system (Malvern Instruments Ltd, Malvern, UK). This system employs a 633 nm laser and a fixed scattering angle (173°). The intensity of light scattered is measured by using an avalanche photodiode. Protein solutions (0.5 and 6 mg/mL), in 20 mM acetate-borate-phosphate buffer adjusted at pH 6, were first passed through a 0.22 μm filter (Millipore, USA), centrifuged at 16,000xg for 10 minutes at room temperature, and subsequently loaded into a quartz cuvette prior to measurement. The temperature was raised from 20 to 85°C and the samples were allowed to equilibrate for 5 minutes in each temperature prior to DLS measurements, after which multiple records of the DLS profile were collected. In each case the hydrodynamic radius was obtained from a second order cumulant fit to the intensity auto-correlation function. The marked point where both the size and the intensity start to increase significantly is called the melting point.

### Zeta-Potential

The zeta-potential in Zetasiser nano-ZS was measured using the laser Doppler velocity (LDV) technique at 20.0 ± 0.1°C. In this technique, a voltage was applied across a pair of electrodes placed at both ends of a cell containing the molecule dispersion. Charged molecules are attracted to the oppositely charged electrode, and their velocity is measured and expressed per unit field strength as the electrophoretic mobility (*μ*_*e*_). Then, the zeta-potential is calculated using Henry´s equation [24,25]: *μ*_*e*_ = [2εζF(k*a*)]/3η, where ε is the dielectric constant of water, ζ is the zeta-potential, and F(κ*a*) is a function of the dimensionless parameter κ*a* (Henry´s function); κ^-1^ = (ε*kT*/2*e* ^2^ *I*)^1/2^ is the double layer thickness, *k* is the Boltzmann constant, *T* is the absolute temperature, *e* is the elementary charge, $I=1/2(\sum {}_{i}c_{i}z_{i}^{2})$is the ionic strength, *c*_*i*_ is the ion concentrations, and *a* is the characteristic dimension of the molecule (protein). The value of this function is 1.5 when the molecules are suspended in aqueous solutions (Smoluchowski approximation) and 1 for nonaqueous media (Hückel approximation). The isoelectric point is given by the pH value at which the zeta-potential is approximately zero. In this study, the zeta potential was measured for a fixed protein concentration of 1 mg/mL (20°C) in 20 mM acetate-borate-phosphate buffer adjusted to the different pH values.

### Small-Angle X-ray Scattering (SAXS) Data Collection

The SAXS measurements were performed at the SAXS2 beamline of LNLS (National Synchrotron Light Laboratory, Campinas, Brazil). For SAXS measurements, TpManGH5 was measured at different protein concentrations (1 and 6 mg/mL in 20 mM acetate-borate-phosphate buffer adjusted at different pH values, pH = 8, 6 and 4) and temperatures (T = 20 and 65°C). The samples were passed through a 0.22 μm filter (Millipore, USA) and centrifuged at 16,000xg for 10 minutes at room temperature prior to measurement. The samples were then loaded into a 1 mm path length cell made of two thin parallel mica windows. The sample temperature was controlled during the measurements. The wavelength of the incoming monochromatic X-ray beam was λ = 1.55 Å and the sample-to-detector distance was set a 0.95 m, providing an *q* (scattering vector) interval from 0.01 to 0.32 Å^-1^, where *q* = 4πsin(θ)/ λ and θ is half the scattering angle. Two successive frames of 300 s were recorded for each sample to monitor radiation damage and beam stability. Buffer scattering was recorded before each sample scattering. The X-ray patterns were recorded using a two-dimensional CCD detector (MarResearch, USA). The parasitic scattering from air and beam-line windows was subtracted from the total measured intensities. The integration of SAXS patterns were performed with the FIT2D software (www.esrf.eu/computing/scientific/FIT2D) and the curves were scaled by protein concentration. The scattering of water measured on the same sample cell was used to normalize the data to absolute scale. No concentration effects were detected for the samples.

### SAXS Data Analysis

The radii of gyration (*R*_*g*_) were determined by two independent procedures: *i*) by the Guinier equation *I*(*q*) = *I*(0).exp[(-*q* ^2^.*R*_*g*_^2^)/3], *q* < 1.3/*R*_*g*_, and *ii*) by the indirect Fourier transform method using the GNOM package (www.emblhamburg.de/biosaxs) [18,21,22]. The distance distribution function *P*(r) was also evaluated with GNOM software and the maximum diameter (*D*_*max*_) was obtained.

### SAXS *ab initio* Modeling

Dummy residue models (DRM) were calculated from the experimental SAXS data by *ab initio* procedures implemented in GASBOR package (www.embl-hamburg.de/biosaxs) [19]. The low-resolution models obtained, in each case, were compared with each other by the use of the DAMAVER [20] procedure and the most representative model for the whole set was used. The resolution (R) was estimated from the equation R = 2π/*q*_max_. The CRYSOL package [32] (www.embl-hamburg.de/biosaxs) was used to generate theoretical scattering curve from the three-dimensional X-ray crystallographic structure of TpManGH5 (PDB 3PZ9). *R*_*g*_ and *D*_*max*_ were determined with the same package. The three-dimensional X-ray crystallographic structure of TpManGH5 and *ab initio* DRM were superimposed with de SUPCOMB package [38]. Superposition Figures were generated by the PyMOL program (www.pymol.org).

### Structural Characterization through Molecular Dynamics Simulations

A better representation of the three-dimensional structure for the different scenarios of pH were obtained using molecular dynamics simulations of all-atoms Structure Based Models (SBM) [28–30]. The topology files for the simulations were generated using SMOG web server (http://smog-server.org) [39] and the equations of motions integrated through GROMACS 4.0.7 package [40]. SBM is a suitable choice due to the structural information provided by the crystallographic structure (PDB id: 3PZ9), allowing a satisfactory conformational searching [29,41]. The total energy of the system is given as a function of the current conformation in comparison (relative) to the native by the following equation:

$$E_{total}\;=\underset{bonds}{\sum }\varepsilon_{r}{\left(r-r_{0}\right)}^{2}+\underset{angles}{\sum }\varepsilon_{\theta }{\left(\theta -\theta_{0}\right)}^{2}\;+\;\sum_{impropers\;}\varepsilon_{\vartheta }{\left(\vartheta -\vartheta_{0}\right)}^{2}+\underset{backbone\;}{\sum }\varepsilon_{BB}\left\{\;\left[1-\text{cos }\left(\varphi -\varphi_{0}\right)\right]+\frac{1}{2}\left[1-\cos\;3\left(\varphi -\varphi_{0}\right)\right]\;\right\}\;\;+\;\underset{sidechain\;}{\sum }\varepsilon_{SC}\left\{\;\left[1-\cos\left(\varphi -\varphi_{0}\right)\right]+\frac{1}{2}\left[1-\cos\;3\left(\varphi -\varphi_{0}\right)\right]\;\right\}\;\;+\;\underset{contacts\;}{\sum }\varepsilon_{LJ}\left[{\left(\frac{\delta_{ij}}{d}\right)}^{12}-2{\left(\frac{\delta_{ij}}{d}\right)}^{6}\right]\;+\;\underset{repulsive\;}{\sum }\varepsilon_{NC}{\left(\frac{\delta_{NC}}{d}\right)}^{12}$$

The values of the parameters with index *0* are obtained from the X-ray crystallographic structure (PDB id: 3PZ9). They are related to the differences in the distance of the covalent bonds (*r* and *r*_*0*_), the angle formed by three consecutive atoms (*θ* and *θ*_*0*_), the planar or improper dihedral angles (*υ* and *υ*_*0*_) and the dihedral angles (*φ* and *φ*_*0*_). The pair distance between the angles *i* and *j* in the contact map/map of interactions in the native structure is *δ*_*ij*_ and the distance between the atoms *i* and *j* in the current conformation is *d*. Atom pairs not included in the contact map are considered repulsive, i.e. non-contacts, with *δ*_*NC*_ *= 2*.*5 Å*. Conveniently, the initial map of contact is defined using the Shadow algorithm [28]. The characterization of the conformational changes stimulated by pH variations were evaluated by doing modifications in the original contact map, and simulated under different temperatures. The modifications in the contact map consist in remove interactions involving 4 regions (loop 1: Y88-A105; loop 2: L195-S206; loop 3: A231-K245 and loop 4: L352-G372, using the PDB 3PZ9 as index) of the protein separately, one-by-one, performing an independent simulations to each one for all 15 possible combinations. The energetic contributions are given as a function of the contact energy *ε*_*LJ*_ *= 1*, in reduced units. The values of ε_*BB*_ and ε_*SC*_ to a ratio equals 2, and the relation *ε*_*LJ*_ by *ε*_*BB*_ plus *ε*_*SC*_ (dihedrals) also equals 2 [30]. The other parameters, *ε*_*r*_, *ε*_*θ*_, *ε*_*υ*_ and *ε*_*NC*_ are defined respectively 10*ε*_*LJ*_ */Å*, *20ε*_*LJ*_ */rad* ^*2*^, *10ε*_*LJ*_ */rad* ^*2*^ and *0*.*01ε*_*LJ*_. The simulations were carried out using Langevin dynamics (sd integrator) having the initial velocities randomly chosen, periodic boundary conditions and using a time step of 0.0005 *ps* for a total of 40,0000,000 steps for each simulation.

### Structural Comparison of the Simulated Conformations and SAXS Data

Theoretical intensities (*I^theory^*) were calculated for the conformations obtained from the simulations and compared with the experimental intensities (*I^experiment^*) obtained by SAXS using the program CRYSOL 2.8 (ATSAS package 2.5.1–1) [32]. The parameter χ^2^ was employed to analyze the agreement between theoretical and experimental values, and it is defined by:

$$\chi^{2}=\frac{1}{N_{q}}\left[\frac{I^{theory}-I^{experiment}}{\gamma \left(q\right)}\right]$$

where, *γ (q)* is the standard deviation, *N*_*q*_ is the number of experimental points and *q* is the scattering vector. Low values of χ^2^ represent a better agreement between the computational and experimental result. The conformations of TpManGH5 obtained from the simulations and *ab initio* DRM were superimposed also using SUPCOMB package [38], and Figures were generated with PyMOL (www.pymol.org).

## Supporting Information

S1 Fig SAXS data collected for TpManGH5 at different concentrations. (A) Experimental SAXS curves of the TpManGH5 at pH 8 and 20°C. (B) Experimental SAXS curves of the TpManGH5 at pH 6 and 20°C. (C) Experimental SAXS curves of the TpManGH5 at pH 4 and 20°C. (D) Experimental SAXS curves of the TpManGH5 at pH 6 and 65°C. Open black squares: 1 mg/mL. Open red circles: 6 mg/mL. Insets: Guinier plots, the curves have been offset for clarity.
(TIF)

S2 Fig SAXS data collected for TpManGH5 at 6 mg/mL and different exposure times. (A) Experimental SAXS curves of the TpManGH5 at pH 8 and 20°C. (B) Experimental SAXS curves of the TpManGH5 at pH 6 and 20°C. (C) Experimental SAXS curves of the TpManGH5 at pH 4 and 20°C. (D) Experimental SAXS curves of the TpManGH5 at pH 6 and 65°C. Open red circles: 300 s. Open black circles: 600 s.
(TIF)

S3 Fig Low-resolution models for TpManGH5 at 20°C and different pH values. (**A**) Molecular envelope of TpManGH5 in solution at pH 8 obtained by GASBOR package. (**B**) Molecular envelope of TpManGH5 in solution at pH 6. (**C**) Molecular envelope of TpManGH5 in solution at pH 4. In all the cases, the center and right structures were rotated y axis-90° and x axis-90° in relation to the left structure.
(TIF)

S4 Fig Kratky plots at 20°C and different pH values. (**A**) Kratky plots (*q* ^2^.I(*q*) versus *q*) generated from the SAXS data collected for TpManGH5 at pH 8 (solid blue line), 6 (solid black line) and 4 (solid red line). The solid green line represent the Kratky plot generated from the computed scattering curve based on the X-ray crystallographic structure of TpManGH5 (PDB 3PZ9). (**B**) Kratky plots generated from the SAXS data collected at pH 8 and 4 overlaid to make the differences between the curves.
(TIF)

S5 Fig Circular dichroism (CD) spectroscopy. Effect of pH on the secondary structure of TpManGH5 at 75°C monitored by far-UV CD spectroscopy. The pH values were 4 (black dash line), 6 (black solid line) and 8 (gray solid line).
(TIF)

S6 Fig DLS pH experiments with TpManGH5 at 20°C. The size distribution by intensity for purified TpManGH5 (1 mg/mL) where DLS runs were conducted at pH 8 (blue line), pH 6 (green line) and 4 (red line).
(TIF)

S7 Fig Gel electrophoresis under denaturing conditions (15% SDS-PAGE). Coomassie-stained showing TpManGH5 after purification. Lane 1, molecular mass standards. Lane 2, TpManGH5.
(TIF)

S8 Fig Low-resolution models for TpManGH5 at pH 6 and different temperatures. (**A**) Molecular envelope of TpManGH5 in solution at 20°C obtained by GASBOR package. (**B**) Molecular envelope of TpManGH5 in solution at 65°C. In both cases, the center and right structures were rotated y axis-90° and x axis-90° in relation to the left structure.
(TIF)

S9 Fig Measure of the local energetic frustration. The Frustratometer Web Server [31] (http://lfp.qb.fcen.uba.ar/embnet/frustra_lf.php) was employed to calculate the TpManGH5 local energetic frustration using the crystallographic structure (PDB 3PZ9). The residue index is defined by the PDB and it is presented in the axis. The favorable pair interactions are represented by filled black squares (■) while frustrated (non-favorable) contact pairs are showed by filled red circles (●). The removed interactions for the different simulations were based on Frustratometer results and on the invariance of the secondary structure under de different pH scenarios, evaluated through circular dichroism (CD) experiments. These regions are indicated by the number 1, 2, 3 and 4 in orange and light gray. The inset Figure shows a cartoon representation of the TpManGH5 backbone in gray. The red and green lines show contacts favorable and frustrated, respectively, being solid lines direct interactions between the residues and dashed lines water-mediated interactions. Energetic frustrations are related to more flexible regions of the enzyme, since these local interactions cannot be properly satisfied. The amount of non-favorable interactions indicates regions were interactions may be removed from the contact map to allow a more complete conformational searching. It is important emphasize that the Structure Based Models (SBM) employed for the simulations do not include charges, playing this approach an important role to generate theoretical scattering curves in a good agreement to the experimental SAXS curves. The loops where the interactions were removed in the different simulations are presented in orange (loop 1) and black (loop 2, loop 3 and loop 4). The models are constructed removing the interactions involving each loop and all possible combination of loops.
(TIFF)

S10 Fig Conformational space explored through SB molecular simulations. Free Energy profile for the simulations where Lennard-Jones interactions between the loop 1 (residues Y88-A105) and the remaining part of the protein were removed. The calculation is performed using the Weighted Histogram Method (WHAM) [42] employing all the temperatures for this model construction (interactions in the loop 1 turned off). The radius of gyration (R_g_*) and the distance between the centers of mass of the Active Site (W134, E198, R200, E235, H283 and W284) [10] and the loop 1 (Distance AS-Loop1) were calculated using GROMACS analysis tools [40]. These reactions coordinates are the best to illustrate the large variety of conformations analyzed. Since the goal of this molecular dynamics is generate tridimensional structures to be compared to the experimental data, the lowest free energy value do not be understood as the most probable conformation experimentally in solution, i.e. the simulations are only one approach to generate conformations that the protein can assume and compare these conformations to the experimental data. Thus, the analysis of the temporal evolution (trajectory) obtained by the simulations employed are not relevant to describe conformational changes or folding/refolding mechanisms initially. The Free Energy is presented in reduced units of energy (ε_LJ_) by the color bar [30]. The green squares numbered by the pH value indicate the position of the conformations with the best agreement with the experimental data (lowest χ^2^). The χ^2^ for all conformations were calculated using CRYSOL [32].
(TIFF)

S1 Table Conformational searching through flexible models. The table presents the lower χ^2^ found for each one of the 14 model constructions.
(PDF)

S2 Table Distance between active site CA atoms. The residues were identified using a structure of TpManGH5 in complex with β-D-glucose (PDB id: 3PZI), where the distances between ligand and residue are lower than 5Å.
(PDF)

S3 Table Estimative of the pKa obtained from PROPKA 3.0. The evaluation was performed using the representative conformations for each scenario on its pH. The protonation of the residue is obtained using the pKa values and the Henderson-Hasselbalch equation.
(PDF)
