# Supplementary figures and images for: Conformational Changes in a Hyperthermostable Glycoside Hydrolase: Enzymatic Activity Is a Consequence of the Loop Dynamics and Protonation Balance
Source: PLoS One. 2015 Feb 27;10(2):e0118225. doi: 10.1371/journal.pone.0118225 (PMC4344334; doi:10.1371/journal.pone.0118225)

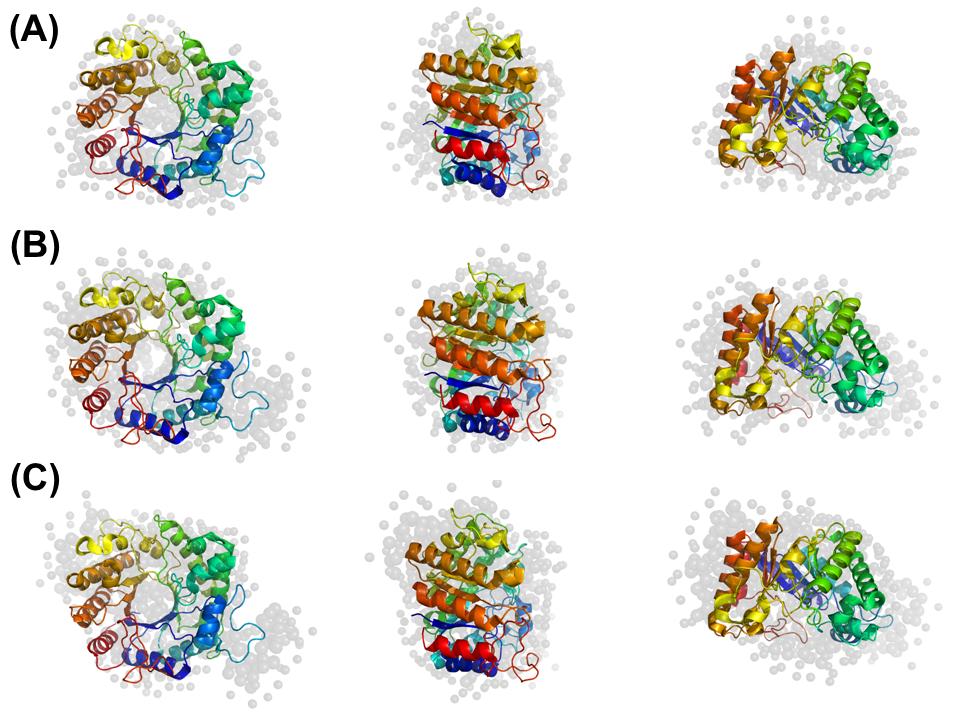

Supplement: S3 Fig — (A) Molecular envelope of TpManGH5 in solution at pH 8 obtained by GASBOR package. (B) Molecular envelope of TpManGH5 in solution at pH 6. (C) Molecular envelope of TpManGH5 in solution at pH 4. In all the cases, the center and right structures were rotated y axis-90° and x axis-90° in relation to the left structure. (TIF) [file pone.0118225.s003.tif]

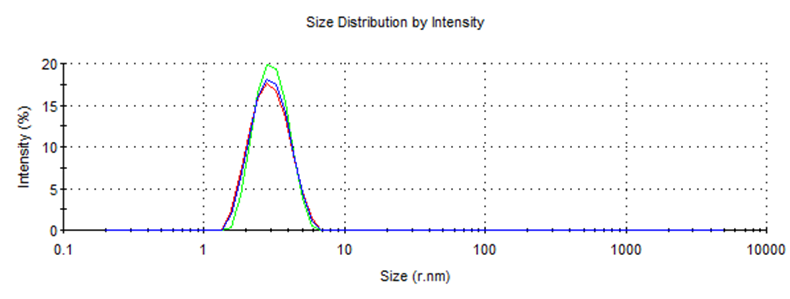

Supplement: S6 Fig — The size distribution by intensity for purified TpManGH5 (1 mg/mL) where DLS runs were conducted at pH 8 (blue line), pH 6 (green line) and 4 (red line). (TIF) [file pone.0118225.s006.tif]

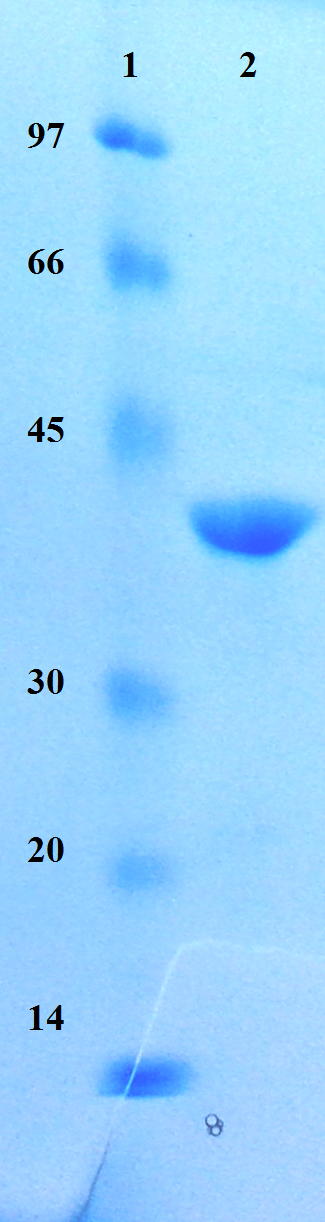

Supplement: S7 Fig — Coomassie-stained showing TpManGH5 after purification. Lane 1, molecular mass standards. Lane 2, TpManGH5. (TIF) [file pone.0118225.s007.tif]

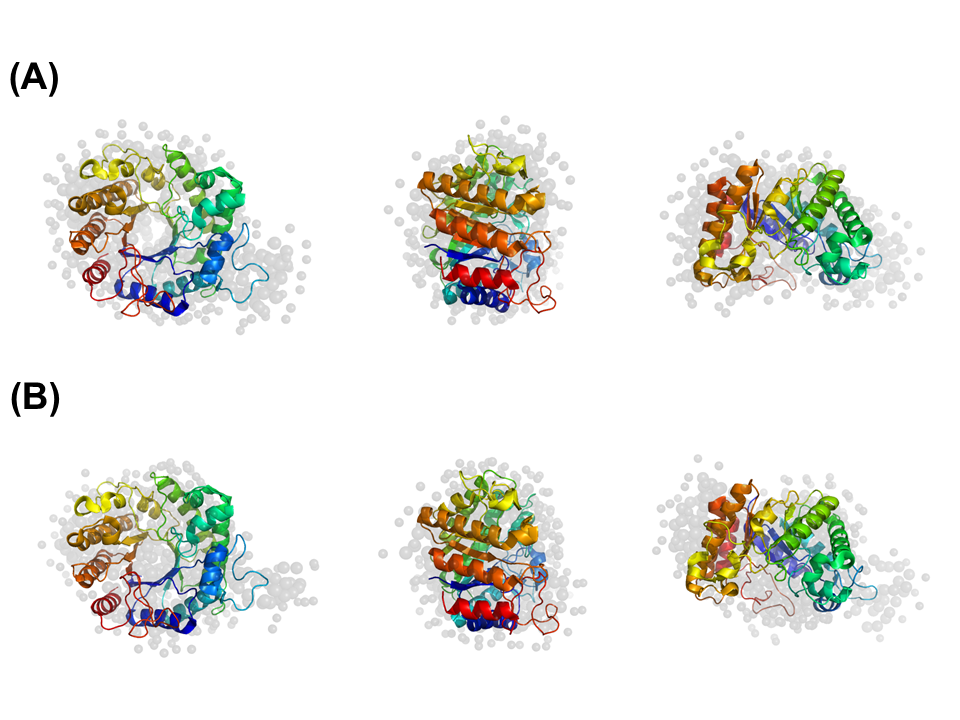

Supplement: S8 Fig — (A) Molecular envelope of TpManGH5 in solution at 20°C obtained by GASBOR package. (B) Molecular envelope of TpManGH5 in solution at 65°C. In both cases, the center and right structures were rotated y axis-90° and x axis-90° in relation to the left structure. (TIF) [file pone.0118225.s008.tif]

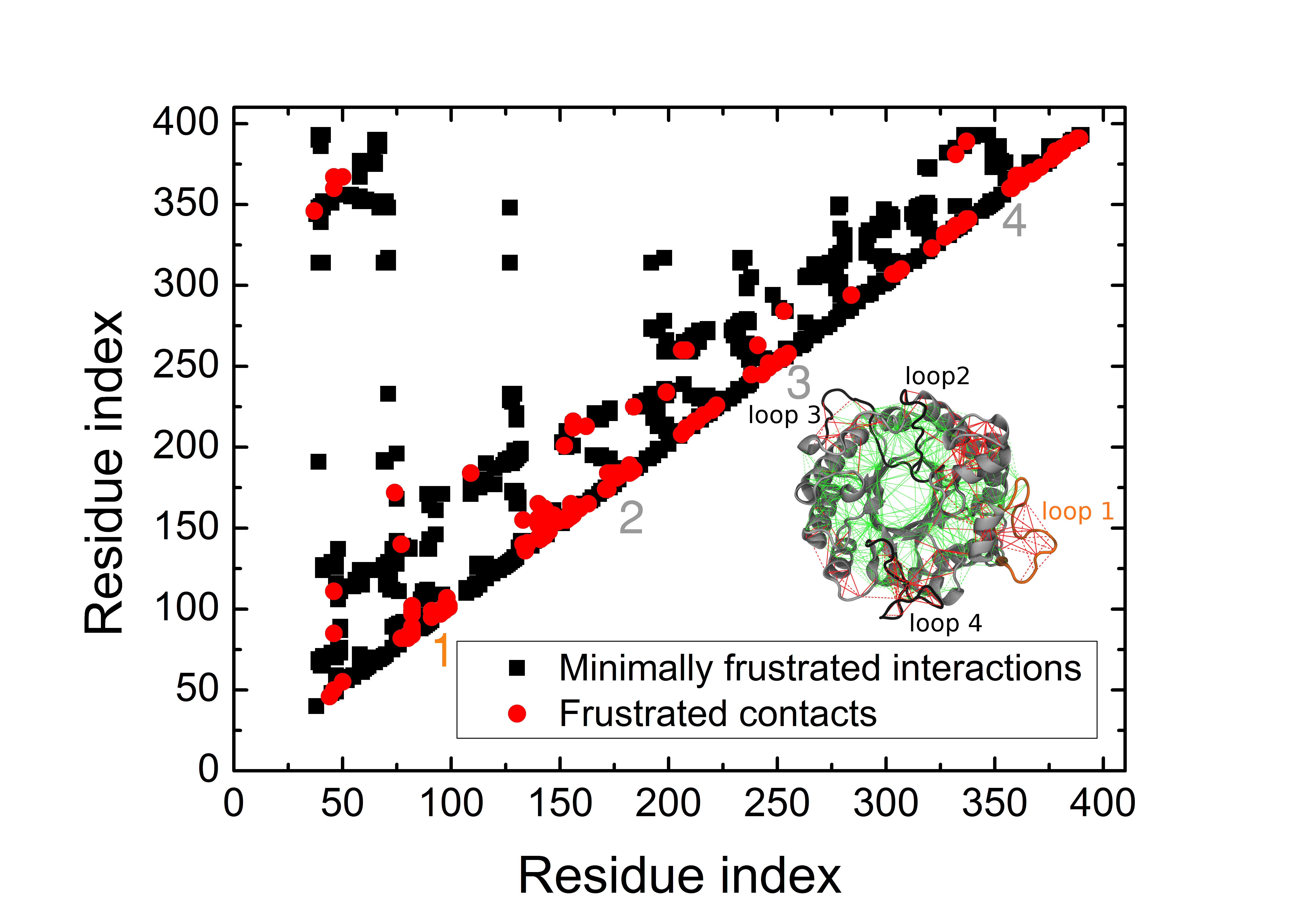

Supplement: S9 Fig — The Frustratometer Web Server [31] (http://lfp.qb.fcen.uba.ar/embnet/frustra_lf.php) was employed to calculate the TpManGH5 local energetic frustration using the crystallographic structure (PDB 3PZ9). The residue index is defined by the PDB and it is presented in the axis. The favorable pair interactions are represented by filled black squares (■) while frustrated (non-favorable) contact pairs are showed by filled red circles (●). The removed interactions for the different simulations were based on Frustratometer results and on the invariance of the secondary structure under de different pH scenarios, evaluated through circular dichroism (CD) experiments. These regions are indicated by the number 1, 2, 3 and 4 in orange and light gray. The inset Figure shows a cartoon representation of the TpManGH5 backbone in gray. The red and green lines show contacts favorable and frustrated, respectively, being solid lines direct interactions between the residues and dashed lines water-mediated interactions. Energetic frustrations are related to more flexible regions of the enzyme, since these local interactions cannot be properly satisfied. The amount of non-favorable interactions indicates regions were interactions may be removed from the contact map to allow a more complete conformational searching. It is important emphasize that the Structure Based Models (SBM) employed for the simulations do not include charges, playing this approach an important role to generate theoretical scattering curves in a good agreement to the experimental SAXS curves. The loops where the interactions were removed in the different simulations are presented in orange (loop 1) and black (loop 2, loop 3 and loop 4). The models are constructed removing the interactions involving each loop and all possible combination of loops. (TIFF) [file pone.0118225.s009.tiff]

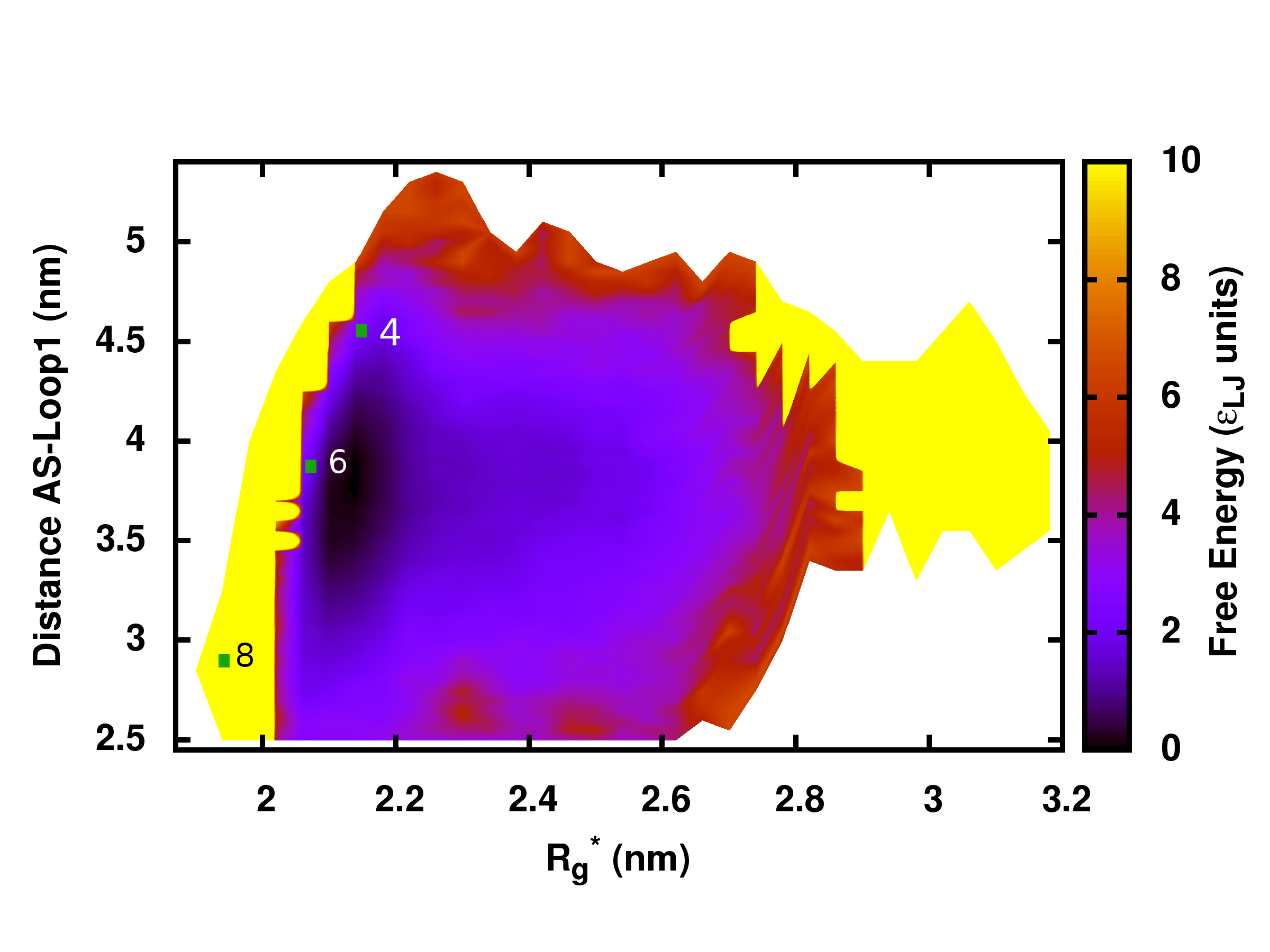

Supplement: S10 Fig — Free Energy profile for the simulations where Lennard-Jones interactions between the loop 1 (residues Y88-A105) and the remaining part of the protein were removed. The calculation is performed using the Weighted Histogram Method (WHAM) [42] employing all the temperatures for this model construction (interactions in the loop 1 turned off). The radius of gyration (Rg*) and the distance between the centers of mass of the Active Site (W134, E198, R200, E235, H283 and W284) [10] and the loop 1 (Distance AS-Loop1) were calculated using GROMACS analysis tools [40]. These reactions coordinates are the best to illustrate the large variety of conformations analyzed. Since the goal of this molecular dynamics is generate tridimensional structures to be compared to the experimental data, the lowest free energy value do not be understood as the most probable conformation experimentally in solution, i.e. the simulations are only one approach to generate conformations that the protein can assume and compare these conformations to the experimental data. Thus, the analysis of the temporal evolution (trajectory) obtained by the simulations employed are not relevant to describe conformational changes or folding/refolding mechanisms initially. The Free Energy is presented in reduced units of energy (εLJ) by the color bar [30]. The green squares numbered by the pH value indicate the position of the conformations with the best agreement with the experimental data (lowest χ2). The χ2 for all conformations were calculated using CRYSOL [32]. (TIFF) [file pone.0118225.s010.tiff]
