# Supplementary material for: Conformational Changes in a Hyperthermostable Glycoside Hydrolase: Enzymatic Activity Is a Consequence of the Loop Dynamics and Protonation Balance
Source: PLoS One. 2015 Feb 27;10(2):e0118225. doi: 10.1371/journal.pone.0118225 (PMC4344334; doi:10.1371/journal.pone.0118225)
Supplement: S1 Table — The table presents the lower χ2 found for each one of the 14 model constructions. (PDF) [file pone.0118225.s011.pdf]

Table S1: Conformational searching through flexible models. The table presents the lower χ^2 found for each one of the 14 model constructions.

	Agreement between curves		
**Loop interactions removed**	**pH 4**	**pH 6**	**pH 8**
		lowest χ^2	
**Shadow**	6.1	11.9	3.2
**1**	2.5	2.9	3.2
**2**	8.3	10.4	3.2
**3**	8.1	10.5	3.2
**4**	7.3	9.4	3.2
**1 and 2**	6.8	9.2	3.2
**1 and 3**	5.4	7.3	3.2
**1 and 4**	5.5	8.5	3.2
**2 and 3**	8.4	9.3	3.2
**2 and 4**	8.1	10	3.2
**3 and 4**	7.8	10.3	3.2
**1, 2 and 3**	5.7	7.7	3.2
**1, 2 and 4**	5.7	7.3	3.2
**1, 3 and 4**	5.3	7	3.2
**2, 3 and 4**	7.2	9.1	3.2
**1, 2, 3 and 4**	5.5	6.4	3.2
**Reference (PDB id: 3PZ9)**	11.9	18.9	3.2
