# Supplementary material for: Conformational Changes in a Hyperthermostable Glycoside Hydrolase: Enzymatic Activity Is a Consequence of the Loop Dynamics and Protonation Balance
Source: PLoS One. 2015 Feb 27;10(2):e0118225. doi: 10.1371/journal.pone.0118225 (PMC4344334; doi:10.1371/journal.pone.0118225)
Supplement: S2 Table — The residues were identified using a structure of TpManGH5 in complex with β-D-glucose (PDB id: 3PZI), where the distances between ligand and residue are lower than 5Å. (PDF) [file pone.0118225.s012.pdf]

Table S2: Distance between active site CA atoms. The residues were identified using a structure of TpMan in complex with β-D-glucose (PDB id: 3PZI), where the distances between ligand and residue are lower than 5 Å.

Pairs	Distances between CA atoms (Å)			Distance between CB atoms (Å)		
	**pH 8**	**pH 6**	**pH 4**	**pH 8**	**pH 6**	**pH 4**
**TRP:134-GLU:198**	7.8	7.3	8.4	6.6	6.3	7.5
**TRP:134-ARG:200**	9.3	9.9	9.8	9.3	9.6	9.8
**TRP:134-GLU:235**	12.6	13	13.7	11.4	12	12.5
**TRP:134-TRP:253**	18.3	19	18.9	17.1	18	18.1
**TRP:134-TYR:280**	15.7	16.3	16.3	13	13.8	13.7
**TRP:134-HIS:283**	14.8	14.4	15.4	12.2	11.9	12.8
**TRP:134-TRP:284**	15.7	15.5	17	14.9	14.8	16.2
**GLU:198-ARG:200**	6.4	6.4	6.1	6.8	6.8	6.4
**GLU:198-GLU:235**	5.3	6	5.7	5.2	6	5.4
**GLU:198-TRP:253**	14.8	15.4	15.3	14.2	15	15.1
**GLU:198-TYR:280**	12	12.6	12.5	9.9	10.7	10.3
**GLU:198-HIS:283**	13.5	13.1	14	11.1	10.8	11.4
**GLU:198-TRP:284**	13.3	13.1	14.3	11.8	11.8	12.7
**ARG:200-GLU:235**	8.7	8.6	9	7.8	7.5	7.6
**ARG:200-TRP:253**	12.3	12.8	13.1	10.8	11.8	11.9
**ARG:200-TYR:280**	15.5	16	16	13.7	14.3	13.9
**ARG:200-HIS:283**	15.6	15.4	15.9	13.6	13.5	13.3
**ARG:200-TRP:284**	14.3	14.2	15.4	12.7	12.7	13.3
**GLU:235-TRP:253**	12.3	12.8	13.2	11.2	11.7	12.6
**GLU:235-TYR:280**	9.3	10	10.1	8.2	9.3	9.1
**GLU:235-HIS:283**	12.4	12.7	13.1	10.5	11.1	11
**GLU:235-TRP:284**	11.3	11.5	12.4	8.9	9.3	10.1
**TRP:253-TYR:280**	13.2	13.3	13.6	12.9	13	13.7
**TRP:253-HIS:283**	12.2	12.2	11.6	12.2	12.2	11.5
**TRP:253-TRP:284**	8.7	8.8	8.6	7.9	7.9	8.2
**TYR:280-HIS:283**	5.6	6	5.7	4.4	4.7	4.6
**TYR:280-TRP:284**	6.2	6.3	6.5	5.5	5.4	6.3
**HIS:283-TRP:284**	3.7	3.5	3.6	5.5	5.3	5.2

**Sum of distances**	**320.8**	**326.1**	**335.2**	**289.3**	**297.2**	**304.2**
**Average**	11.5	11.6	12	10.3	10.6	10.9
**Standard deviation**	3.7	3.8	3.9	3.2	3.3	3.4
