# Supplementary material for: Conformational Changes in a Hyperthermostable Glycoside Hydrolase: Enzymatic Activity Is a Consequence of the Loop Dynamics and Protonation Balance
Source: PLoS One. 2015 Feb 27;10(2):e0118225. doi: 10.1371/journal.pone.0118225 (PMC4344334; doi:10.1371/journal.pone.0118225)
Supplement: S3 Table — The evaluation was performed using the representative conformations for each scenario on its pH. The protonation of the residue is obtained using the pKa values and the Henderson-Hasselbalch equation. (PDF) [file pone.0118225.s013.pdf]

Table S3: Estimative of the pKa obtained from PROPKA 3.0. The evaluation was performed using the representative conformations for each scenario on its pH. The protonation of the residue is obtained using the pKa values and the Henderson-Hasselbalch equation.

	pKa-pKmodel			pKa			pKmodel	$[A^-]/[HA]$		
Residue	pH 4	pH 6	pH 8	pH 4	pH 6	pH 8		pH 4	pH 6	pH 8
37	-0.12	-0.06	-0.14	7.88	7.94	7.86	8	0.0001	0.0115	1.3804
38	-0.17	-0.61	-0.3	12.33	11.89	12.2	12.5	0	0	0.0001
45	-0.14	5.83	2.12	9.86	15.83	12.12	10	0	0	0.0001
46	1.41	3.16	2.71	11.41	13.16	12.71	10	0	0	0
48	-2.35	-3.69	-3.15	4.15	2.81	3.35	6.5	0.7079	1548.817	44668.37
49	5.65	2.07	4.56	15.65	12.07	14.56	10	0	0	0
50	0.01	-2.01	-0.08	10.51	8.49	10.42	10.5	0	0.0032	0.0038
53	0.21	0.15	0.62	12.71	12.65	13.12	12.5	0	0	0
56	-1.13	0.09	0.12	2.67	3.89	3.92	3.8	21.3796	128.8249	12022.64
60	0.17	1.16	0.71	4.67	5.66	5.21	4.5	0.2138	2.1878	616.595
63	0.23	0.25	-0.08	12.73	12.75	12.42	12.5	0	0	0
64	0.16	-0.14	0.39	3.96	3.66	4.19	3.8	1.0965	218.7761	6456.542
68	-0.09	-0.34	-0.05	10.41	10.16	10.45	10.5	0	0.0001	0.0035
71	1.4	1.09	3.73	13.9	13.59	16.23	12.5	0	0	0
77	1.61	2.67	4.96	5.41	6.47	8.76	3.8	0.0389	0.3388	0.1738
79	0.22	0.1	-0.75	4.72	4.6	3.75	4.5	0.1905	25.1189	17782.79
81	0.39	0.84	0.56	10.39	10.84	10.56	10	0	0	0.0028
82	-0.05	0.49	2.9	8.95	9.49	11.9	9	0	0.0003	0.0001
83	-0.03	-0.37	1.28	12.47	12.13	13.78	12.5	0	0	0
84	0.17	0.18	-0.78	3.97	3.98	3.02	3.8	1.0715	104.7129	95499.27
85	-0.06	-0.81	0.37	10.44	9.69	10.87	10.5	0	0.0002	0.0013
88	0.13	0.08	0.83	10.13	10.08	10.83	10	0	0.0001	0.0015
90	-0.07	-0.61	-4.02	6.43	5.89	2.48	6.5	0.0037	1.2882	331131.1
92	0.13	0.43	0.76	4.63	4.93	5.26	4.5	0.2344	11.749	549.5406
100	0.04	0.14	0.05	4.54	4.64	4.55	4.5	0.2884	22.9087	2818.382
110	-0.14	0.08	-1.68	4.36	4.58	2.82	4.5	0.4365	26.3027	151356.2
111	-0.38	-1.12	1.43	12.12	11.38	13.93	12.5	0	0	0
113	1.6	1.87	1.07	5.4	5.67	4.87	3.8	0.0398	2.138	1348.963
114	2.02	3.31	2.68	12.02	13.31	12.68	10	0	0	0
118	0.37	1.21	1.25	10.87	11.71	11.75	10.5	0	0	0.0002
120	0	-0.09	0.66	10.5	10.41	11.16	10.5	0	0	0.0007
121	0.07	-0.73	-0.94	4.57	3.77	3.56	4.5	0.2692	169.8244	27542.29
125	-0.46	-0.8	-0.44	10.04	9.7	10.06	10.5	0	0.0002	0.0087
135	-0.13	0.23	-0.37	3.67	4.03	3.43	3.8	2.138	93.3254	37153.5

136	0.45	0.87	0.84	4.25	4.67	4.64	3.8	0.5623	21.3796	2290.868
143	2.94	5.03	7.71	12.94	15.03	17.71	10	0	0	0
145	0	-0.29	-0.02	12.5	12.21	12.48	12.5	0	0	0
151	0.04	0.23	-0.17	6.54	6.73	6.33	6.5	0.0029	0.1862	46.7735
152	-1.33	-0.98	0.21	5.17	5.52	6.71	6.5	0.0676	3.02	19.4984
153	-0.43	-0.53	-1.63	3.37	3.27	2.17	3.8	4.2658	537.0318	676082.9
154	0.07	-0.26	-0.52	3.87	3.54	3.28	3.8	1.349	288.4032	52480.78
156	3.8	5.16	5.58	13.8	15.16	15.58	10	0	0	0
157	0.06	0.18	0.46	12.56	12.68	12.96	12.5	0	0	0
158	0.5	-0.19	-1.06	4.3	3.61	2.74	3.8	0.5012	245.4709	181970.2
159	0.25	0.15	-0.71	4.75	4.65	3.79	4.5	0.1778	22.3872	16218.1
160	-0.09	0.03	0.7	12.41	12.53	13.2	12.5	0	0	0
162	-0.39	1.11	1.74	10.11	11.61	12.24	10.5	0	0	0.0001
163	-1.02	-0.34	0.13	3.48	4.16	4.63	4.5	3.3113	69.1831	2344.228
164	0.24	0.29	1.44	4.74	4.79	5.94	4.5	0.182	16.2181	114.8153
165	3.34	3.48	4.19	13.34	13.48	14.19	10	0	0	0
166	0.04	-0.53	0.63	10.54	9.97	11.13	10.5	0	0.0001	0.0007
167	-0.05	0.66	0.03	10.45	11.16	10.53	10.5	0	0	0.003
168	5.03	4.1	5.22	15.03	14.1	15.22	10	0	0	0
175	-0.22	-0.61	-0.29	6.28	5.89	6.21	6.5	0.0052	1.2882	61.6595
179	0.6	0.24	1.49	10.6	10.24	11.49	10	0	0.0001	0.0003
184	4.4	3.91	6.93	14.4	13.91	16.93	10	0	0	0
185	0.02	-0.18	-0.06	12.52	12.32	12.44	12.5	0	0	0
186	0.05	0.18	0.05	4.55	4.68	4.55	4.5	0.2818	20.893	2818.382
187	1.83	0.01	-1.19	6.33	4.51	3.31	4.5	0.0047	30.9029	48977.89
194	5.15	2.09	-0.13	9.65	6.59	4.37	4.5	0	0.257	4265.796
198	-2.15	-0.59	3.32	2.35	3.91	7.82	4.5	44.6684	123.0269	1.5136
200	0.29	-0.17	2.13	12.79	12.33	14.63	12.5	0	0	0
201	2.29	2.58	3.14	11.29	11.58	12.14	9	0	0	0.0001
202	0.06	-0.14	0.09	4.56	4.36	4.59	4.5	0.2754	43.6516	2570.395
204	-0.13	-0.78	-2.43	3.67	3.02	1.37	3.8	2.138	954.9926	4265797
205	0	-0.57	-0.42	10.5	9.93	10.08	10.5	0	0.0001	0.0083
212	0.11	0.25	0.18	4.61	4.75	4.68	4.5	0.2455	17.7828	2089.297
215	-0.54	-0.37	-0.21	9.96	10.13	10.29	10.5	0	0.0001	0.0051
216	0.47	-0.84	-1.64	4.97	3.66	2.86	4.5	0.1072	218.7761	138038.5
220	0.25	-0.39	0.51	10.25	9.61	10.51	10	0	0.0002	0.0031
222	-0.29	0.01	0.25	10.21	10.51	10.75	10.5	0	0	0.0018
225	2.06	0.01	-1.27	5.86	3.81	2.53	3.8	0.0138	154.8817	295121.1
228	-1.84	0.52	0.23	4.66	7.02	6.73	6.5	0.2188	0.0955	18.6209
234	2.65	2.08	3.08	6.45	5.88	6.88	3.8	0.0035	1.3183	13.1826
235	4.51	6.19	3.02	9.01	10.69	7.52	4.5	0	0	3.02

241	2.53	0.64	4.74	12.53	10.64	14.74	10	0	0	0
242	-0.09	0.08	0.04	4.41	4.58	4.54	4.5	0.389	26.3027	2884.032
245	-0.54	-0.08	-0.02	9.96	10.42	10.48	10.5	0	0	0.0033
247	0.14	0.5	0.35	10.14	10.5	10.35	10	0	0	0.0045
250	-0.6	1.18	-1.92	3.9	5.68	2.58	4.5	1.2589	2.0893	263026.8
252	0.27	0.38	0.08	4.77	4.88	4.58	4.5	0.1698	13.1826	2630.269
255	4.26	3.01	5.29	14.26	13.01	15.29	10	0	0	0
262	-0.42	0.87	0.04	3.38	4.67	3.84	3.8	4.1687	21.3796	14454.39
264	-0.4	-0.93	-0.21	10.1	9.57	10.29	10.5	0	0.0003	0.0051
265	0.41	-0.79	0.12	10.91	9.71	10.62	10.5	0	0.0002	0.0024
270	0.05	0.19	0.02	4.55	4.69	4.52	4.5	0.2818	20.4174	3019.952
273	-0.15	0.62	-0.54	3.65	4.42	3.26	3.8	2.2387	38.0189	54954.06
278	0.14	1.78	-5.63	6.64	8.28	0.87	6.5	0.0023	0.0052	13489632
280	8.05	7.48	9.03	18.05	17.48	19.03	10	0	0	0
283	-0.42	-0.25	-0.71	6.08	6.25	5.79	6.5	0.0083	0.5623	162.181
289	0.11	-0.81	-0.34	4.61	3.69	4.16	4.5	0.2455	204.1738	6918.312
291	0.7	1	1.04	10.7	11	11.04	10	0	0	0.0009
297	-0.13	-0.59	0.07	10.37	9.91	10.57	10.5	0	0.0001	0.0027
300	0.23	0.88	0.79	4.73	5.38	5.29	4.5	0.1862	4.1687	512.8615
301	2.01	1.92	1.04	5.81	5.72	4.84	3.8	0.0155	1.9055	1445.439
302	-4.09	-5.82	-3.17	2.41	0.68	3.33	6.5	38.9045	208929.7	46773.52
304	0.08	-0.02	-0.03	10.58	10.48	10.47	10.5	0	0	0.0034
307	-0.13	-0.19	-0.23	10.37	10.31	10.27	10.5	0	0	0.0054
308	0.06	0.13	-0.02	4.56	4.63	4.48	4.5	0.2754	23.4423	3311.311
311	-0.71	-0.07	-1.21	9.79	10.43	9.29	10.5	0	0	0.0513
316	1.02	4.35	8.09	5.52	8.85	12.59	4.5	0.0302	0.0014	0
317	5.91	1.26	1.68	10.41	5.76	6.18	4.5	0	1.7378	66.0694
318	5.15	4	3.48	15.15	14	13.48	10	0	0	0
322	-0.11	-0.28	-0.18	10.39	10.22	10.32	10.5	0	0.0001	0.0048
328	-0.29	0.48	0.97	12.21	12.98	13.47	12.5	0	0	0
332	2.46	1.42	2.28	12.46	11.42	12.28	10	0	0	0.0001
333	0.16	0.08	2.28	12.66	12.58	14.78	12.5	0	0	0
337	0.56	0.96	0.15	4.36	4.76	3.95	3.8	0.4365	17.378	11220.19
340	0.15	0.18	0.26	10.15	10.18	10.26	10	0	0.0001	0.0055
341	-0.09	0.53	0.54	3.71	4.33	4.34	3.8	1.9498	46.7735	4570.88
345	0.55	0.38	0.35	4.35	4.18	4.15	3.8	0.4467	66.0694	7079.456
357	0.2	0.73	-1.21	4.7	5.23	3.29	4.5	0.1995	5.8884	51286.14
360	-0.5	0.43	-1.35	3.3	4.23	2.45	3.8	5.0119	58.8844	354813.6
361	-0.12	-0.07	-0.18	12.38	12.43	12.32	12.5	0	0	0
362	-0.9	-1.48	-1.92	2.9	2.32	1.88	3.8	12.5893	4786.302	1318256
363	0.17	1.12	-0.46	4.67	5.62	4.04	4.5	0.2138	2.3988	9120.109

364	0.36	0.17	1.28	12.86	12.67	13.78	12.5	0	0	0
366	2.97	0.34	0.55	12.97	10.34	10.55	10	0	0	0.0028
367	2.16	9.04	6.38	12.16	19.04	16.38	10	0	0	0
369	0.57	-0.68	-0.19	4.37	3.12	3.61	3.8	0.4266	758.5778	24547.11
370	0.44	1.62	1.97	10.44	11.62	11.97	10	0	0	0.0001
371	1.86	2.57	0.72	5.66	6.37	4.52	3.8	0.0219	0.4266	3019.952
374	-0.38	-0.42	0.25	12.12	12.08	12.75	12.5	0	0	0
378	-0.47	-0.82	-1.84	3.33	2.98	1.96	3.8	4.6774	1047.129	1096478
379	0.25	0.01	0.12	4.05	3.81	3.92	3.8	0.8913	154.8817	12022.64
382	4.12	1.41	-0.86	8.62	5.91	3.64	4.5	0	1.2303	22908.66
384	0.22	-0.04	-0.24	4.72	4.46	4.26	4.5	0.1905	34.6737	5495.406
387	0.14	-0.12	0.15	12.64	12.38	12.65	12.5	0	0	0
388	0.06	0.24	-1.21	4.56	4.74	3.29	4.5	0.2754	18.197	51286.14
389	0.96	0.83	2.06	10.96	10.83	12.06	10	0	0	0.0001
391	-0.38	-0.2	-0.1	10.12	10.3	10.4	10.5	0	0.0001	0.004
393	-0.08	0.18	0.03	3.12	3.38	3.23	3.2	7.5858	416.8693	58884.36
